# Morbidity associated with schistosomiasis in adult population of Chókwè district, Mozambique

**DOI:** 10.1371/journal.pntd.0012738

**Published:** 2024-12-16

**Authors:** João Tiago Serra, Carina Silva, Mohsin Sidat, Silvana Belo, Pedro Ferreira, Natália Ferracini, Daniel Kaminstein, Ricardo Thompson, Claúdia Conceiçao

**Affiliations:** 1 Institute of Hygiene and Tropical Medicine, IHMT, NOVA University, Lisbon, Portugal; 2 Global Health and Tropical Medicine, GHTM, Associate Laboratory in Translation and Innovation Towards Global Health, LA-REAL, IHMT, NOVA University, Lisbon, Portugal; 3 Health & Technology Research Center, H&TRC, School of Health Technology, ESTeSL, Polytechnical Institute of Lisbon, Lisbon, Portugal; 4 Centro de Estatística e Aplicações, CEAUL, Universidade de Lisboa, Lisbon, Portugal; 5 Faculty of Medicine, University Eduardo Mondlane, Maputo, Mozambique; 6 Medical College of Georgia at Augusta University, Augusta, Georgia, United States of America; 7 Chókwè Health Research and Training Center, National Institute of Health, Chókwè, Mozambique; University of Cartagena, COLOMBIA

## Abstract

**Background:**

Mozambique is one of the countries with the highest prevalence of schistosomiasis, although there is little data on the prevalence of disease and associated morbidity in the adult population. This study aimed to describe and characterize the morbidity associated with schistosomiasis in the adult population of Chókwè district and to explore the use of anamnestic questionnaires and urine dipsticks, as well as point-of-care ultrasound for urinary related findings, to better characterize disease prevalence and morbidity.

**Methodology:**

Between April and October 2018, we conducted a cross-sectional study embedded within the Chókwè Health Research and Training Centre. Data were collected on sociodemographic variables, signs and symptoms for schistosomiasis and water related activities. Infection status was determined by urine filtration, Kato-Katz thick smear and DNA detection. Point-of care urinary tract ultrasonography was performed to assess structural morbidity associated with *Schistosoma haematobium* infection. Multivariate logistic regression was used to search for associations between risk factors, signs and symptoms, infection status and ultrasound abnormalities.

**Principal findings:**

Our study included 1033 participants with a median age of 34 years old. The prevalence of *Schistosoma haematobium*, *Schistosoma mansoni* and ultrasound detected urinary tract abnormalities were 11.3% (95% CI 9.5%-13.4%), 5.7% (95% CI 4.3%-7.5%) and 37.9% (95% CI 34.8%-41.2%), respectively. Of the 37.9% with urinary tract abnormalities, 14.5% were positive for *Schistosoma haematobium*. Reported hematuria in the last month (p = 0.004, aOR 4.385) and blood in the urine dipstick (p = 0.004, aOR 3.958) were markers of *Schistosoma haematobium* infection. Reporting lower abdominal pain (p = 0.017, aOR 1.599) was associated with ultrasound abnormalities.

**Conclusion:**

Using microscopy and DNA analysis for both *Schistosoma haematobium* and *Schistosoma mansoni* in conjunction with urinary ultrasound abnormalities gives us several insights into correlations between disease prevalence (microscopic and anatomical) and demographic details in a high-risk population.

## Author summary

Schistosomiasis is one of the most common parasitic diseases in the world, mainly affecting people living in sub-Saharan Africa. Mozambique is one of the countries with the highest prevalence of the disease. Chronic urinary schistosomiasis in the adult population can lead to hematuria, anemia, urinary tract lesions and eventually death from renal failure and bladder cancer. The characteristics of the chronic form of disease in the adult population of Mozambique is unknown. A cross-sectional, community-based study was conducted to assess current morbidity and the risk factors for schistosomiasis among the adult population of Chókwè district, Mozambique. The prevalence of urinary schistosomiasis was 11.3%, intestinal schistosomiasis was 5.7% and urinary tract ultrasound abnormalities were 37.9%. Of the 37.9% with ultrasound abnormalities, 14.5% were positive for *Schistosoma haematobium*. In this study, reporting hematuria in the last month and blood in the urine were found to be diagnostic markers of urinary schistosomiasis, and reporting lower abdominal pain was associated with ultrasound abnormalities. The high prevalence of anatomical urinary tract ultrasound abnormalities suggests that the disease has an important impact on the long-term health of the adult population.

## Introduction

Schistosomiasis, also known as Bilharzia, is an infectious disease classified as one of the 20 Neglected Tropical Diseases (NTDs), and remains a public health problem in several parts of the world, particularly in Africa, where 91% of infected people live [1]. Of the approximately 21 known species of *Schistosoma*, only 6 are pathogenic to humans, namely *S*. *haematobium*, *S*. *mansoni*, *S*. *japonicum*, *S*. *mekongi*, *S*. *intercalatum* and *S*. *guineensis* [2,3]. The immunology of schistosomiasis shows that the eggs, rather than the adult worms, are responsible for the pathological effects of infection [4]. The majority of clinical disease manifestations occur at body sites of increased egg accumulation, such as the bladder wall and ureters in case of S. *haematobium* infection and the liver, portal system and intestine for *S*. *mansoni* [5,6]. The classic sign of urinary schistosomiasis is the presence of blood in the urine. In advanced cases, kidney damage and fibrosis of the bladder and ureters can occur [7]. Bladder cancer is a reported complication in later stages of *S*. *haematobium* infection and the parasite is therefore classified as carcinogen [8,9]. Intestinal schistosomiasis can cause abdominal pain, diarrhea and presence of blood in the stools [7]. The diagnostic algorithm for schistosomiasis includes important anamnestic questions, specific clinical signs and supporting laboratory investigation [10].

Mozambique is one of the countries with the highest prevalence of schistosomiasis [11]. Several studies have reported a wide geographical variation in the prevalence of schistosomiasis in the country, depending on the location and population studied [12–27]. The most recent nationwide study estimated a global prevalence of 53.5% for geohelminths, 47% for *S*. *haematobium* and 1% for *S*. *mansoni* among individuals aged 7–22 years old [28]. The morbidity and mortality associated with schistosomiasis are grossly underestimated in most countries and long-term morbidity mainly affects adults resulting in chronic diarrhea, malabsorption, renal failure and bladder cancer [29–31]. The economic and social impact of the disease results in reduced ability to work and a reduction in available family income [32]. In an attempt to quantify the clinical morbidity associated with schistosomiasis in sub-Saharan Africa, hematuria associated with *S*. *haematobium* infection was estimated to occur in 70 million individuals and dysuria in 32 million. Vesical wall lesions and hydronephrosis were estimated to affect 106 and 18.9 million people, respectively. *S*. *mansoni* infection caused diarrhea in 780000 people, blood in the stool in 4.4 million and hepatomegaly in 8.5 million. Mortality due to end-stage renal disease and upper gastrointestinal bleeding accounted for 150000 and 130000 deaths per year, respectively [33,34].

Mozambique began mass treatment of school-aged children with Praziquantel in 2010. By 2011, approximately 32.8% of the country had been covered [35]. Although treatment of adults in hyperendemic areas (>50%) and at-risk groups in areas with prevalence below 50% was planned, it was not consistently achieved due to material and human resource difficulties, as well as a lack of data [35]. Detection of NTD-related morbidity is a major challenge, given the limited availability of laboratory and other diagnostic facilities in most health facilities throughout the country, and also due to the lack of qualified staff to diagnose complications. Reducing the morbidity associated with schistosomiasis by 2020 was one of the goals of the Mozambican Integrated National Plan for the Control of Neglected Tropical Diseases 2013–2017 [35].

Ultrasound has demonstrated significant diagnostic capabilities in assessing morbidity due to various infectious diseases in tropical settings [36–39]. The World Health Organization (WHO) has recognized its good safety, flexibility and cost-effectiveness in assisting in the diagnosis of morbidity. In the specific case of urinary schistosomiasis, ultrasound has been shown to be a good alternative to other invasive procedures such as cystoscopy [40]. The use of bedside ultrasound by clinicians to facilitate urgent assessment or to support invasive procedures, has become increasingly popular and it`s being incorporated into medical training programs worldwide [36]. In areas highly endemic for *S*. *haematobium* infection, urinary ultrasound findings such as hydronephrosis, hydroureter, bladder wall thickening, and bladder wall polyps and/or masses, have been considered a good proxy for schistosomiasis related morbidity [41].

An important gap in the prevalence of disease associated morbidity for schistosomiasis in the adult population of Mozambique remains. The aim of this study was to describe and characterize the morbidity associated with schistosomiasis in the adult population of Chókwè district and to explore the correlation with anamnestic questionnaire results and microbiologic findings. Ultrasound was used to assess anatomical abnormalities and results of these findings are mentioned briefly in this paper, but will be more completely described in an upcoming publication.

## Methods

### Ethical considerations

Administrative approval was obtained from the Gaza Provincial Health Directorate and the protocol was submitted to the Institutional Bioethics Committee of the National Institute of Health and the Mozambican National Bioethics Committee for Health (83/CNBS/2017). Final administrative approval was deferred by the Minister of Health of Mozambique. The Chókwè Consultative Committee was consulted and the District Health, Women and Social Action Service, the District Government and the Municipal Council of the town of Chókwè were informed as part an ongoing collaboration. The conditions for the transfer of biological material between the Chókwè Health Research and Training Center (CITSC) and the Institute of Hygiene and Tropical Medicine from NOVA University were established under a biological material transfer agreement between the two institutions.

Depending on their age and spoken language (Portuguese or Changana), each individual was given an informed consent form. In case of acceptance, individuals over 18 years old signed a written consent form or, in case of illiteracy, printed their fingerprint, in which case the signature of a witness outside the research team was required. In the case of minors (15 to 17 years old), in addition to the fingerprint or written consent of the minor, the consent of the legal guardian was required, following a procedure similar to that described above in the case of individuals over 18 years old. All consent forms were completed in duplicate, with one copy kept by the participant and the other by the research team.

A preparatory meeting was held in each of the units prior to field activities in order to present and explain the study. At the end of the study, medical treatment was provided to all participants with schistosomiasis or other diagnosed intestinal parasitic infection. If any ultrasound abnormalities were detected that required additional treatment, participants were referred to the nearest health facility for further treatment.

### Study area and population

**Fig 1:**
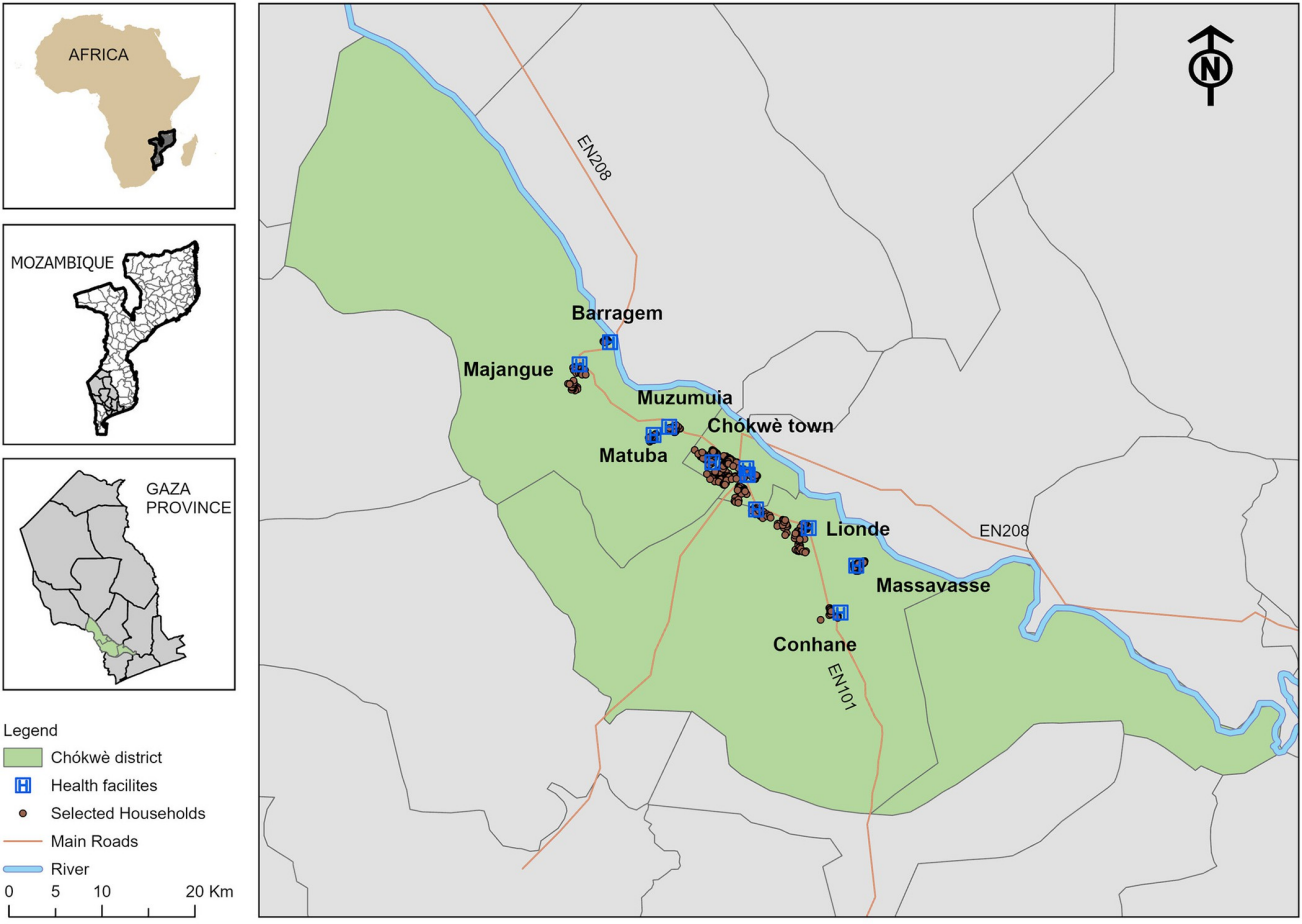
Map of the Chókwè district, health facilities and study included households. The study took place in Chókwè district, Gaza province, south of Mozambique. Map created using ArcGIS Pro 3.3.0. Base layer map provided by GADM—University of California-Davis, Mapcruzin, Stanford University, available from: https://icedrive.net/s/50XyM9uso5.

Covering an area of 2466 km^2^, Chókwè is a small and densely populated district (88 inhabitants/Km^2^) in the south of Gaza province, Mozambique (Fig 1). In the 1950s, the construction of a dam allowed the development of a large irrigation area consisting of several canals and ditches. This ecological change allowed for the spread of schistosomiasis and other tropical diseases [42,43].

The Chókwè Health Research and Training Center is a research center of the National Institute of Health of Mozambique established in 2007. This center has been implementing and managing a continuous demographic surveillance system (HDSS) since 2010 and has been part of the “International Network for Demographic Evaluation of the Population and Their Health” (INDEPTH) since 2014 [44]. The HDSS covers a geographical area of about 600 km^2^ and includes 14 administrative units, of which 7 are classified as neighborhoods of the town of Chókwè and 7 as villages in the district. In 2018, 19877 aggregates were active in the HDSS database, representing about 100000 of the 183000 inhabitants of Chókwè district. All individuals registered in the HDSS are identified with a unique, lifelong and non-transferable code that allows the individual to be tracked in the community. In Chókwè district, the prevalence of *S*. *haematobium* infection in school-aged children is estimated to be 35%, but no specific data is available on the prevalence of *S*. *mansoni* infection in the district (personal communication).

### Study design and sampling

We conducted a cross-sectional study of individuals aged 15 years or older (WHO defines school-age as 5–15 for schistosomiasis control strategies), living in households registered in the HDSS database in 2018. In that year, the HDSS registered 19578 households with at least one member aged 14 years or older. A two-stage cluster sampling procedure was used to calculate the sample. For this purpose, the household was considered as the primary sampling unit and the smallest administrative units of the HDSS as the secondary sampling unit. The number of clusters required was determined on the basis of the methodology recommended by the WHO in the Extended Program on Immunization [45]. The selection of 30 clusters was therefore recommended, with the aim of enrolling 30 individuals per cluster (30x30). Considering the smallest administrative units of the HDSS (n = 131) as the secondary sampling unit, but taking into account the variation in the number of households that make up each unit, the probability proportional to population size method was used to standardize the probability of selection for each of the clusters. After systematically selecting 30 HDSS units, the second step was to select 30 individuals in each of the 30 clusters. Therefore, using the household as the primary sampling unit, it was necessary to calculate the number of households required to include at least 30 individuals per cluster. With an average of 2 persons aged 15 or over per household, 13 households would be required to obtain at least 30 persons per cluster. A refusal rate of 20% was also included in the calculation, in line with experience from previous studies conducted by CITSC. The final calculation resulted in 19 households per cluster, which were randomly selected from the HDSS database, resulting in a total of 570 households selected to participate in the study.

For each of the clusters, a list of selected households was generated, containing the name and date of birth of the members as recorded in the HDSS database. However, given the high mobility of the population and the likelihood of outdated data, if none of the members of a household could be found after two attempts, or if their dwelling could not be located, the household was replaced by the nearest one whose members were present. All participants who reported living in the house at the time of the survey were included, regardless of whether they were on the researcher`s list or how long they had lived in the household.

The inclusion and exclusion criteria for the study were applied by the research team members in the field. Inclusion criteria included: age 15 years or older at the time of recruitment; living in the selected household at the time of recruitment; ability to understand and consent. Exclusion criteria were as follows: known or ultrasound documented pregnancy (pregnancy is known to increase rates of hydronephrosis independent of pathology) at the time of recruitment; history of abdominal trauma requiring medical attention in the previous 6 months; history of kidney or bladder surgery, documented by the presence of an abdominal or back scar not attributable to another cause reported by the participant; individuals not located after at least two location attempts; individuals not providing a biological sample.

### Individual and household data

Before starting the work in the selected clusters and aggregates, a pilot study was conducted to test the procedures and adjust the working methodology. The fieldwork was carried out between April and October 2018. The individual questionnaire (S1 Appendix) was developed on the basis of previously used questionnaires, which were further developed to allow the assessment of demographic information, signs, symptoms and water related activities [46–48]. It was administered by members of the research team with the exception of the main researcher. This was not only due to the frequent need for fluency in Changana when interacting with the participant, but also to allow the main researcher to be unaware of the presence of signs and symptoms suggestive of disease when performing the ultrasound. The questionnaire was completed electronically using a tablet. Completion of the individual questionnaire was mandatory for all participants, who were identified only by the individual code assigned in the context of this study. A housing conditions questionnaire (S2 Appendix) based on information previously recorded by the HDSS surveillance system was completed for each of the study households. All participating households were required to complete a housing conditions questionnaire.

### Sample collection and analysis

Urine was collected between 10:00 and 14:00 although it was occasionally necessary to collect outside these hours. Fecal samples were preferentially collected early in the morning. Samples were then placed in a thermal suitcase with cooling devices, transported to the CITSC laboratory and analyzed using standardized quality-controlled techniques, preferably within 24 hours of collection. Urine samples were examined microscopically for *S*. *haematobium* and were also tested for blood, leukocytes, proteins and nitrites [49]. Kato-Katz (KK) thick smears were examined microscopically for *S*. *mansoni*, soil-transmitted helminths and other parasites [50].

For molecular analysis, all urine and fecal samples were centrifuged, the sediment was stored at –20°C and transferred to long-term –80°C storage at the Institute of Hygiene and Tropical Medicine of the NOVA University Laboratory. Genomic DNA extraction was performed using the Speed Tools Tissue DNA kit (Biotools B&M Labs, Madrid, Spain) according to the manufacture’s protocol. For the PCR analysis, specific primers for *S*. *haematobium* and *S*. *mansoni*, were sequenced (Stab Vida, Caparica, Portugal) [51,52].

### Point-of-care ultrasounds

The full details of the ultrasound protocol and image analysis will be detailed in a separate paper. Briefly, all study participants were invited to undergo an abdominal ultrasound using a portable ultrasound system. Ultrasound was not a mandatory part of the study; absence of ultrasound results did not result in the exclusion of a participant from other aspects of study analysis. Ultrasound examinations followed a predetermined protocol which was developed based on the WHO recommendations for the assessment of structural changes associated with *S*. *haematobium* infection, incorporating modifications suggested in more recent literature (S3 Appendix) [53–55].

### Statistical analysis

Participants were considered positive for schistosomiasis infection if at least one of the diagnostic methods (parasitological or molecular test) gave a positive result. Urine dipstick results were classified as negative and positive. Participants with at least one positive item on the ultrasound protocol were classified as having a urinary tract abnormality.

The statistical methods used included descriptive statistics, bivariate analysis using odds ratio and multivariate logistic regression modelling. Descriptive statistics were used to measure relative frequencies and percentages of the variables. Bivariate analysis was performed to identify the factors associated with *S*. *haematobium* infection, *S*. *mansoni* infection and ultrasound abnormalities to be included in the multivariate logistic regression for risk factor analysis. Chi-squared tests or Fisher’s exact test, when the conditions for applicability were not met, were used to determine the association between variables. Variables with significance at values 0.05 in the univariate test and/or with biological plausibility were selected for multivariate logistic analysis, to identify the risk factors for *S*. *hematobium*, *S*. *mansoni* and ultrasound abnormalities. Results were reported as crude odds ratios (cOR) with 95% confidence intervals (CI) and adjusted odds ratios (aORs) with 95% confidence intervals, together with the test for significance. Data analysis was performed using IBM SPSS (version 29.0) and *p*-values less than 0.05 were considered significant.

## Results

### Study participation and compliance

**Fig 2:**
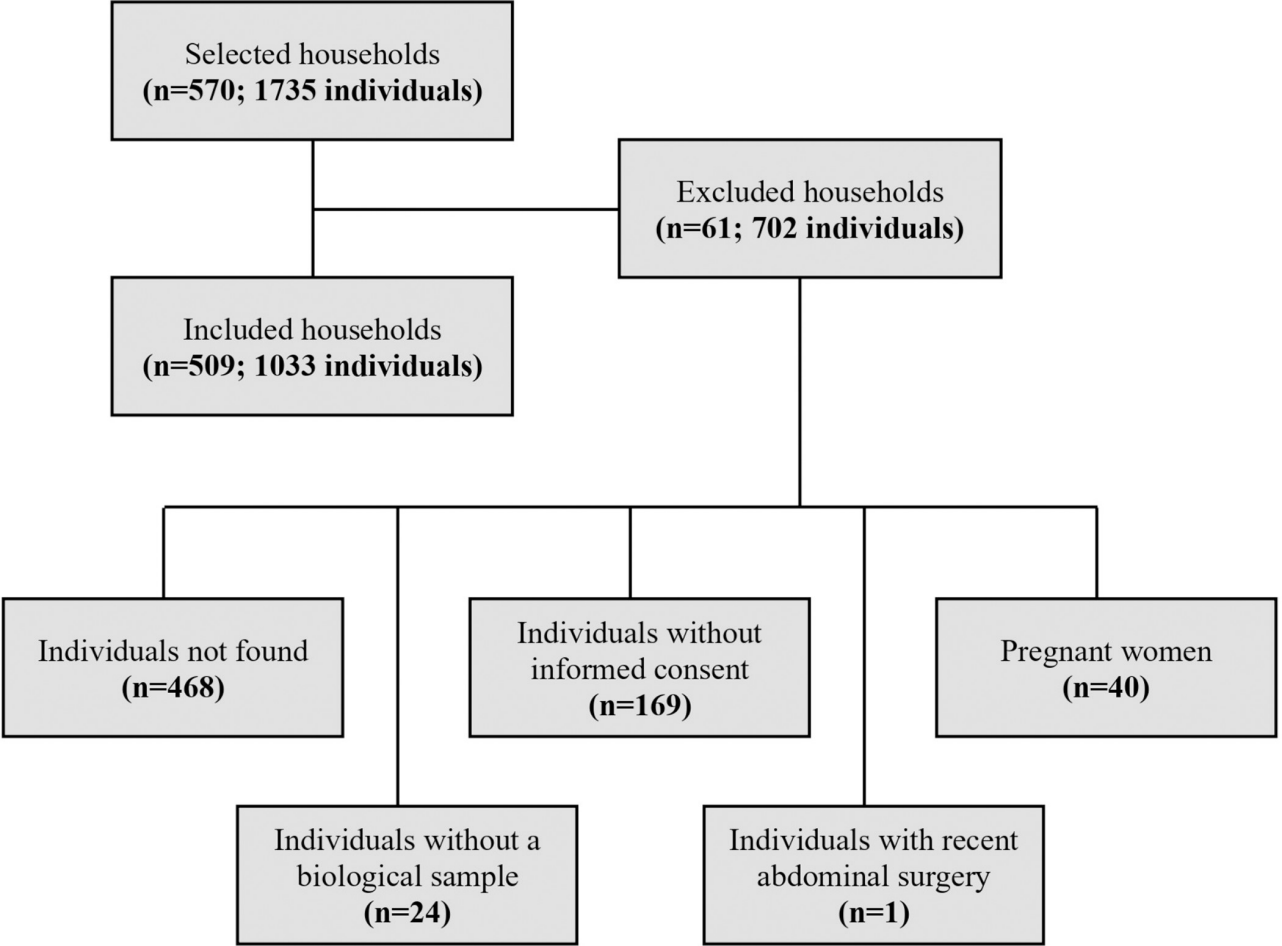
Flow chart of participation according to inclusion and exclusion criteria established for the study. Participants were recruited between April and October 2018.

The final study sample consisted in 1033 individuals aged 15 years and over (Fig 2).

### Sociodemographic and household`s characteristics

Sociodemographic characteristics are summarized in Table 1. Most participants were female (71.7%) and ranged in age from a minimum of 15 years to a maximum of 98 years. The median age was 34 (IQR 32) years.

**Table 1:** Sociodemographic characteristics.

Population characteristics		n (%)
Sex (n = 1033)	Female	741 (71.7)
	Male	292 (28.3)
Age groups, years (n = 1033)	15–24	347 (33.6)
	25–34	180 (17.4)
	35–44	164 15.9)
	45–54	105 (10.2)
	55–64	133 12.9)
	≥ 65	104 (10.1)
Marital status (n = 1033)	Single	342 (33.1)
	Married or cohabiting	482 (46.7)
	Divorced or separated	53 (5.1)
	Widower	156 (15.1)
Education (n = 1032)	None	118 (11.4)
	Primary	557 (54.0)
	Secondary	342 (33.1)
	Higher education and post-graduation	15 (1.5)
Occupation (n = 1032)	Unemployed or retired	226 (21.9)
	Student	187 (18.1)
	Agriculture and fishing	392 (38.0)
	Industry and construction	59 (5.7)
	Business and services	145 (14.1)
	Other	23 (2.2)
Place of birth (n = 1033)	Chókwè district	615 (59.5)
	Another location	418 (40.5)
Length of residency (n = 1031)	< 20 years	318 (30.8)
	≥ 20 years	713 69.2)

Table 2 shows the characteristics of the households. Most of them (61.1%) were located in the semi-urban area of Chókwè town and the rest (38.9%) in the rural area of the district. The average number of persons living in the same house was 6.4, with a minimum of 1 and a maximum of 20. The average number of persons under the age of 15 living in the same house was 2.6, ranging from 0 to 13.

**Table 2:** Household’s characteristics.

Household characteristics		n (%)
Number of people living in the same house (n = 1033)	< 6	457 (44.2)
	≥ 6	576 (55.8)
Water source (n = 1033)	Piped water	921 (89.2)
	Well	112 (10.8)
Water source location (n = 1033)	Inside home	31 (3.0)
	Inside the backyard	747 (72.3)
	Outside the house or yard	75 (7.3)
	At the neighbor’s house	113 (10.9)
	Other	67 (6.5)
Bathroom/Sanitation facilities (n = 1033)	No	49 (4.7)
	Toilet	165 (16.0)
	Improved latrine	240 (23.2)
	Basic latrine	566 (54.8)
	Other	13 (1.3)
Electricity (n = 1033)	Yes	836 (80.9)
Kitchen (n = 1033)	Yes	808 (78.2)
Cooking method (n = 1015)	Firewood	581 (57.2)
	Electricity	13 (1.3)
	Coal	420 (41.4)
	Other	1 (0.1)

### Health-related characteristics

The most commonly reported symptom was fever (39.3%), followed by lower abdominal pain (35.1%) and dysuria (30.9%). The proportion of people positive for *S*. *haematobium* infection reporting hematuria in the last month was 45.5% and for those with a documented ultrasound abnormality was 64.1% (Table 3).

**Table 3:** Health-related characteristics according to *Schistosoma* infection and ultrasound abnormality.

Signs, symptoms and clinical background	Total n (%)	*Schistosoma haematobium* n (%)	*Schistosoma mansoni* n (%)	Ultrasound abnormality n (%)
Hematuria, any time in live	298/989 (30.1)	47/298 (15.8)	14/274 (5.1)	112/273 (41.0)
Hematuria, last month	44/943 (4.5)	20/44 (45.5)	2/40 (5.0)	25/39 (64.1)
Dysuria, last month	315/1019 (30.9)	41/315 (13.0)	19/292 (6.5)	135/287 (47.0)
Difficulty emptying the bladder, last month	257/1020 (25.2)	34/257 (13.2)	18/235 (7.7)	108/243 (44.4)
Abdominal pain, last month	312/1025 (30.4)	31/312 (9.9)	19/283 (6.7)	105/276 (38.0)
Lower abdominal pain, last month	363/1033 (35.1)	48/363 (13.2)	23/331 (6.9)	148/330 (44.8)
Diarrhea, last month	182/1026 (17.7)	24/182 (13.2)	9/170 (5.3)	69/171 (40.4)
Blood in stool, last month	81/960 (8.4)	13/81 (16.0)	5/78 (6.4)	38/74 (51.4)
Worms or parasites in stool, last month	34/941 (3.6)	6/34 (17.6)	3/33 (9.1)	13/31 (41.9)
Fever, last month	406/1032 (39.3)	43/406 (10.6)	21/369 (5.7)	142/366 (38.8)
Malaria, anytime in the past	891/1001 (89.0)	108/891 (12.1)	47/800 (5.9)	290/790 (36.7)
Schistosomiasis, anytime in the past	283/854 (33.1)	36/283 (12.7)	11/262 (4.2)	104/259 (40.2)
Filariasis, anytime in the past	2/1028 (0.2)	0/2 (0.0)	0/2 (0.0)	0/2 (0.0)
Worms or intestinal parasites, anytime in the past	183/833 (22)	22/183 (12.0)	17/172 (8.1)	66/170 (38.8)
Onchocerciasis, anytime in the past	0/1033 (0.0)	-	-	-
Tuberculosis, anytime in the past	137/1027 (13.3)	14/137 (10.2)	7/127 (5.5)	56/123 (45.5)
HIV infection, anytime in the past	188/1022 (18.4)	23/188 (12.2)	11/273 (6.4)	63/169 (37.3)
Schistosomiasis treatment, anytime in the past	160/875 (18.3)	21/160 (13.1)	4/151 (2.6)	59/147 (40.1)
Intestinal parasites treatment, anytime in the past	84/852 (9.9)	8/84 (9.5)	2/80 (2.5)	26/76 (34.2)

### Infection, urine dipstick and ultrasound data

The prevalence of infection by egg and/or DNA detection was 11.3% (95% CI 9.5%-13.4%) for *S*. *haematobium* and 5.7% (95% CI 4.3%-7.5%) for *S*. *mansoni* (Table 4). There were 3 cases of co-infection with both species. Infection intensity for *S*. *haematobium* infection was light (<50eggs/10mL) in 34/38 (89.5%) and heavy (≥50 eggs/10mL) in 4/38 (10.5%) of cases. For intestinal schistosomiasis, the only samples positive for *S*. *mansoni* eggs had a light infection intensity (<100epg). The proportion of people infected with *S*. *haematobium* was higher in rural areas (16.9% vs 7.8%), but the results for *S*. *mansoni* infection were slightly higher in semi-urban areas (5.4% vs 6.2%). Parasitological results for other intestinal parasites are presented in S4 Appendix.

**Table 4:** *Schistosoma* infection according to laboratorial results.

Parasite	Parasitological results n (%)	Molecular results n (%)	Parasitological and/or molecular results n (%)
*Schistosoma haematobium*	38/1033 (3.7)	103/1033 (10.0)	117/1033 (11.3)
*Schistosoma mansoni*	2/922 (0.2)	51/922 (4.9)	53/922 (5.7)

Blood in the urine was the most common urine dipstick finding (19%), followed by proteinuria in 14.2% of cases. With regard to blood in the urine, 5.3% (39/741) of women were excluded because they were menstruating or had menstruated in the previous 48 hours. Of those reporting blood in the urine, 24.6% were infected with *S*. *haematobium* and 44.9% had an ultrasound abnormality (Table 5).

**Table 5:** Urine dipstick findings according to *Schistosoma* infection and ultrasound abnormality.

Urinalysis	Total n (%)	*Schistosoma haematobium* n (%)	Ultrasound abnormality n (%)
Blood in urine	189/994 (19.0)	52/189 (27.5)	72/170 (45.0)
Proteinuria	147/1033 (14.2)	34/147 (23.1)	52/125 (41.6)
Leukocytes	60/1033 (5.8)	13/60 (21.7)	23/56 (41.1)
Nitrites	38/1033 (3.7)	9/38 (23.7)	22/36 (61.1)

Of the 912 ultrasound scans performed, 37.9% (346/912) were classified as abnormal, of which 14.5% (50/346) were confirmed positive for *S*. *haematobium*. An inferior urinary tract abnormality was found in 11.8% (108/912) and an upper urinary tract abnormality in 29.7% (271/912). More specific details of ultrasound abnormalities will be detailed in a future publication. Assuming that a urinary tract ultrasound abnormality is an indirect measure of chronic schistosomiasis in the present epidemiological context, the overall prevalence of structural morbidity associated with schistosomiasis is 37.9% (95% CI 34.8%-41.2%).

### Water activities

Regarding the usual water activities (Table 6), 43.7% reported some contact with water from rivers, streams or lakes. The most common water activity was using water from rivers, streams or lakes for agricultural activities (29.6%), followed by crossing rivers, streams or lakes (28.7%). More than 90% of participants reported using soap for washing (99.3%), doing laundry (98.1%) and washing dishes (95.5%), but less than 50% used soap for hand washing (46.9%).

**Table 6:** Water activities according to *Schistosoma* infection and ultrasound abnormality.

Activity	Total n (%)	*Schistosoma haematobium* n (%)	*Schistosoma mansoni* n (%)	Ultrasound abnormality n (%)
To have contact with water from rivers, streams or lakes	451/1033 (43.7)	70/451 (15.5)	32/409 (7.8)	153/403 (38.0)
To do the laundry with water from rivers, streams or lakes	91/1030 (8.8)	19/91 (20.9)	11/80 (13.8)	33/79 (41.8)
To wash dishes with water from rivers, streams or lakes	62/1023 (6.1)	10/62 (16.1)	6/59 (10.2)	21/52 (40.4)
To wash yourself with water from rivers, streams or lakes	176/1031 (17.1)	29/176 (16.5)	15/160 (9.4)	60/160 (37.5)
To wash the children with water from rivers, streams or lakes	34/1025 (3.3)	5/34 (14.7)	5/33 (15.2)	13/30 (43.3)
To swim in water from rivers, streams or lakes	40/1031 (3.9)	10/40 (25.0)	2/36 (5.6)	18/36 (50.0)
To cross rivers, streams or lakes	296/1032 (28.7)	43/296 (14.5)	20/274 (7.3)	110/268 (41.0)
To cook with water from rivers, streams or lakes	59/1027 (5.7)	7/59 (11.9)	7/57 (12.3)	18/53 (34.0)
To fish with a net in rivers, streams or lakes	7/1033 (0.7)	0/7 (0.0)	1/7 (14.3)	4/7 (57.1)
To fish with a hook in rivers, streams or lakes	19/1033 (1.8)	5/19 (26.3)	0/15 (0.0)	7/16 (43.8)
To use water from rivers, streams or lakes for agriculture activities	305/1030 (29.6)	46/305 (15.1)	20/277 (7.2)	106/272 (39.0)
To use water from rivers, streams or lakes for religious activities	8/1031 (0.8)	2/8 (25.0)	0/8 (0.0)	1/8 (12.5)
To use water from rivers, streams or lakes for other activities	119/1030 (11.6)	21/119 (17.6)	14/109 (12.8)	43/111 (38.7)
To use soap to do the laundry	969/988 (98.1)	110/969 (11.4)	51/864 (5.9)	330/854 (38.6)
To use soap to wash dishes	887/929 (95.5)	101/887 (11.4)	48/797 (6.0)	311/790 (39.4)
To use soap to wash hands	484/1032 (46.9)	49/484 (10.1)	24/437 (5.5)	148/427 (34.7)
To use soap to wash yourself	1026/1033 (99.3)	113/1026 (11.0)	53/915 (5.7)	344/905 (38.0)

### Association of *Schistosoma* infection with risk factors and morbidity parameters

All variables were analyzed for bivariate association with *S*. *haematobium* infection, *S*. *mansoni* infection and ultrasound abnormalities (S5, S6 and S7 Appendices), but most of them dropped out when included in a multivariate model. Age (p = 0.003), time of residency in Chókwè (p<0.001), occupation (p = 0.009) and the number of people living in the same house less then 15 years old (p = 0.004) were the sociodemographic characteristics associated with *S*. *haematobium* infection (Table 7). Reporting hematuria anytime in life (p = 0.004) and hematuria in the last month (p<0.001), were the only symptoms associated with infection. Of all the water activities examined, a positive association was found with seven of them (Table 7). After multivariate analysis, the only four variables with statistical significance were: age (p = 0.011, aOR = 0.977, 95% CI 0.959–0.995), students (p = 0.025, aOR = 0.389, 95% CI 0.170–0.890), reporting hematuria in the last month (p = 0.002, aOR = 4.267, 95% CI 1.718–10.596) and blood in urine dipstick (p<0.001, aOR 3.193, 95% CI 1.834–5.560).

**Table 7:** Risk factors and morbidity parameters associated with *Schistosoma haematobium* infection.

Risk factor, sign and symptom		*Schistosoma haematobium*			
		p value^a^	Crude OR (95% CI)	p value^b^	Adjusted OR (95% CI)
Sex (ref: male)		0.651	1.105 (0.716–1.708)	0.950	0.978 (0.490–1.952)
Age		0.003	0.976 (0.964–0.987)	**0.011**	**0.977 (0.959–0.995)**
Occupation	unemployed or retired	0.009	-	-	-
	student		0.617 (0.340–1.120)	**0.025**	**0.389 (0.170–0.890)**
	agriculture and fishing		0.780 (0.488–1.245)	0.051	0.341 (0.115–1.005)
	industry and construction		0.856 (0.374–1.959)	0.771	0.901 (0.445–1.822)
	trade and services		0.236 (0.096–0.575)	0.335	0.519 (0.137–1.969)
	other		-	0.999	-
Time of residency (ref: ≤ 20 years)		<0.001	0.397 (0.268–0.587)	0.058	0.540 (0.285–1.021)
Number of people in the same house less then 15 years old		0.004	1.064 (0.971–1.166)	0.825	0.985 (0.864–1.124)
Hematuria, anytime in life (ref: no)		0.004	1.803 (1.205–2.698)	0.267	1.391 (0.777–2.490)
Hematuria, last month (ref: no)		<0.001	6.944 (3.397–14.198)	**0.002**	**4.267 (1.718–10.596)**
To do the laundry with water from rivers, streams or lakes (ref: no)		0.003	2.265 (1.310–3.914)	0.291	1.576 (0.677–3.666)
To wash yourself with water from rivers, streams or lakes (ref: no)		0.018	1.719 (1.091–2.711)	0.852	1.081 (0.478–2.445)
To swim in water from rivers, streams or lakes (ref: no)		0.011*	2.754 (1.310–5.791)	0.448	0.607 (0.167–2.203)
To cross rivers, streams or lakes (ref: no)		0.040	1.520 (1.016–2.275)	0.374	1.370 (0.684–2.744)
To fish with a hook in rivers, streams or lakes (ref: no)		0.037	2.876 (1.017–8.136)	0.839	0.842 (0.160–4.418)
To use water from rivers, streams or lakes for agriculture activities (ref: no)		0.012	1.662 (1.115–2.576)	0.416	1.316 (0.680–2.547)
To use water from rivers, streams or lakes for other activities (ref: no)		0.022	1.819 (1.085–3.049)	0.879	0.941 (0.429–2.061)
Blood in urine dipstick (ref: no)		<0.001	4.629 (3.065–6.992)	**<0.001**	**3.193 (1.834–5.560)**
Proteinuria in urine dipstick (ref: no)		<0.001	2.911 (1.865–4.543)	0.193	1.547 (0.801–2.987)
Leukocytes in urine dipstick (ref: no)		0.009	2.311 (1.210–4.414)	0.353	1.509 (0.633–3.596)
Nitrites in urine dipstick (ref: no)		0.031*	2.549 (1.175–5.527)	0.765	1.185 (0.389–3.606)
Ultrasound abnormality (ref: no)		0.005	1.823 (1.197–2.777)	0.102	1.513 (0.921–2.483)

ref, reference class

* bivariate analysis using Fisher`s exact test

^a^ bivariate analysis

^b^ multivariate analysis

As shown in Table 8, females (p = 0.035) were more likely to be infected with *S*. *mansoni*. Participants who reported doing laundry (p = 0.004) and using water from rivers, streams or lakes for other activities (p<0.001), were more likely to be infected with *S*. *mansoni*, but only this last activity retained significancy in the multivariate analysis (p = 0.017, aOR 2.452, 95% CI 1.172–5.130).

**Table 8:** Risk factors and morbidity parameters associated with *Schistosoma mansoni* infection.

Risk factor, sign and symptom	*Schistosoma mansoni*			
	p value^a^	Crude OR (95% CI)	p value^b^	Adjusted OR (95% CI)
Sex (ref: male)	0.035	2.234 (1.038–4.807)	0.118	1.863 (0.854–4.065)
Age	0.357	1.003 (0.989–1.018)	0.770	1.002 (0.987–1.018)
To do the laundry with water from rivers, streams or lakes (ref: no)	0.004*	3.025 (1.490–6.141)	0.101	2.235 (0.854–5.845)
To wash yourself with water from rivers, streams or lakes (ref: no)	0.031*	1.968 (1.055–13.672)	0.793	0.891 (0.375–2.116)
To wash the children with water from rivers, streams or lakes (ref: no)	0.034*	3.180 (1.175–8.609)	0.356	1.777 (0.524–6.024)
To cook with water from rivers, streams or lakes (ref: no)	0.040*	2.483 (1.067–5.781)	0.870	1.089 (0.392–3.025)
To use water from rivers, streams or lakes for other activities (ref: no)	<0.001	2.921 (1.530–5.577)	**0.017**	**2.452 (1.172–5.130)**

ref, reference class

* bivariate analysis using Fisher`s exact test

^a^ bivariate analysis

^b^ multivariate analysis

Being born in Chókwè (p = 0.026), reporting hematuria (p = 0.001), dysuria (p<0.001), lower abdominal pain (p = 0.001), nitrites (p = 0.003), blood in urine dipstick (p = 0.034) and *S*. *haematobium* infection (p = 0.005) were all associated with participants having a urinary tract ultrasound abnormality. After multivariate analysis (Table 9), individuals born in Chókwè district (p = 0.007, aOR 0.608, 95% CI 0.423–0.875) and reporting lower abdominal pain in the last month (p = 0.017, aOR 1.599, 95% CI 1.089–2.349) had significantly higher odds of having an ultrasound abnormality.

**Table 9:** Risk factors and morbidity parameters associated with ultrasound abnormalities.

Risk factor, sign and symptom	Ultrasound abnormality			
	p value^a^	Crude OR (95% CI)	p value^b^	Adjusted OR (95% CI)
Sex (ref: male)	0.171	1.235 (0.913–1.672)	0.772	0.940 (0.621–1.424)
Age	0.184	1.000 (0.993–1.007)	0.430	1.004 (0.994–1.014)
Place of birth (ref: Chókwè district)	0.026	0.734 (0.558–0.965)	**0.007**	**0.608 (0.423–0.875)**
Hematuria, last month (ref: no)	0.001	3.088 (1.593–6.261)	0.511	1.340 (0.559–3.214)
Dysuria, last month (ref: no)	<0.001	1.772 (1.331–2.359)	0.126	1.406 (0.909–2.177)
Difficulty emptying the bladder, last month (ref: no)	0.011	1.475 (1.094–1.990)	0.782	1.065 (0.683–1.661)
Lower abdominal pain, last month (ref: no)	0.001	1.577 (1.196–2.079)	**0.017**	**1.599 (1.089–2.349)**
Blood in stool, last month (ref: no)	0.016	1.794 (1.111–2.895)	0.043	1.934 (1.020–3.667)
Schistosomiasis, anytime in the past (ref: no)	0.138	1.264 (0.927–1.723)	0.381	0.789 (0.465–1.340)
Schistosomiasis treatment, anytime in the past (ref: no)	0.396	1.173 (0.812–1.694)	0.163	1.501 (0.848–2.655)
To have contact with water from rivers, streams or lakes (ref: no)	0.988	1.002 (0.765–1.312)	0.212	0.796 (0.556–1.139)
Blood in urine dipstick (ref: no)	0.034	1.452 (1.027–2.055)	0.348	1.237 (0.793–1.930)
Nitrites in urine dipstick (ref: no)	0.003	2.677 (1.351–5.306)	0.053	2.345 (0.987–5.569)
*Schistosoma haematobium* infection (ref: no)	0.005	1.823 (1.197–2.777)	0.279	1.364 (0.778–2.392)

ref, reference class. All bivariate analysis was done using Chi-square test

^a^ bivariate analysis

^b^ multivariate analysis

## Discussion

The objectives of this study were to describe and characterize the morbidity associated with schistosomiasis in the adult population of Chókwè district and to explore the use of anamnestic questionnaires and molecular results to highlight positive correlations. Multivariate analysis showed that younger people, students, people reporting hematuria in the last month or with blood in the urine dipstick had an increased risk of urinary schistosomiasis. Urinary tract ultrasound abnormalities were found in 37.9% of participants, of which 14.5% were positive for *S*. *haematobium*. Individuals reporting lower abdominal pain in the last month had higher chances of having an ultrasound abnormality.

### Infection prevalence in Chókwè district

Our results confirmed the presence of both *S*. *haematobium* (11.3%, 95% CI 9.5%-13.4%) and *S*. *mansoni* (5.7%, 95% CI 4.3%-7.5%) infection among adults registered in the Chókwè HDSS. The only previous data available on the prevalence of schistosomiasis in this area of Mozambique dates back to the 1950s, reporting a prevalence of 37.7% for *S*. *haematobium* and 0.4% for *S*. *mansoni* in indigenous adults, 2.86% for *S*. *haematobium* in the European population, and 68% for *S*. *haematobium* and 2% for *S*. *mansoni* in school-aged children [12,15,16]. A recent study in the Manhiça area, reported a prevalence of *S*. *haematobium* and *S*. *mansoni* in adults of 7.1% and 3.1%, respectively [27]. However, the Ministry of Health reported a *S*. *haematobium* prevalence of 35% among Chókwè school-aged children in 2005–2007. Although there are no specific data on *S*. *mansoni* in Chókwè district, the reported prevalence among school-aged children for Gaza province is 0.8% [28]. The discrepancy between the previous *S*. *haematobium* data and the current actual findings in the adult population (37.7% in 1952 versus 11.3% in 2018) may be due to several reasons.

Firstly, water, sanitation and hygiene conditions, one of the cornerstones of schistosomiasis control, has been steadily improving in Mozambique, contributing to a reduction in schistosomiasis prevalence [56,57]. In our study, 89.2% of people had piped water, most of them inside the house or in the backyard, and 95.3% had a sanitation facility, the majority of which was a basic latrine (54.8%), which is a major improvement compared to the housing conditions of the local population is the 1950s. Secondly, two mass chemotherapy campaigns took place in 2014 and 2016, covering 91% and 90% of school-aged children in Chókwè, respectively (personal communication). These campaigns likely reduced prevalence among school-aged children, thereby reducing transmission rates and decreasing prevalence in the adult population.

### Diagnostic challenges in low prevalence areas

Although parasitological detection of *Schistosoma* remains the cornerstone of schistosomiasis diagnosis, a sharp decline in sensitivity in low endemic areas affect disease assessment [58,59]. The main method used to diagnose urinary schistosomiasis is to filter and concentrate *S*. *haematobium* eggs from urine, followed by microscopic examination. Eggs are generally easy to identify, but these tests tend to have low sensitivity in the adult population, mainly due to reduced egg excretion with chronicity of the disease [60]. The Kato-Katz thick stool smear method, which also relies on microscopy, is still widely used and is the WHO recommended standard for the diagnosis of intestinal schistosomiasis. When the host has a high number of worms, the sensitivity of KK is high because of the large number of eggs excreted, but as with urine filtration for *S*. *haematobium* diagnosis, the accuracy of the test decreases in individuals with mild infections and in regions of low disease prevalence [61,62]. In addition, schistosome egg excretion fluctuates daily, leading to high variability in KK test results due to overdispersal of egg production, especially in cases of mild infections. The exact level of disease impact within a community may be significantly underestimated by the KK method. Therefore, because of their simplicity and cost-effectiveness, KK and urine egg detection techniques are particularly well suited to settings with high schistosome transmission levels, but lack the necessary sensitivity in areas of low endemicity. Our study found a prevalence of *S*. *haematobium* and *S*. *mansoni* of 3.7% and 0.2% by parasitological testing compared with 10% and 4.9% by DNA detection, respectively (Table 4). Our data highlights the need to search for new methods of evaluation that can answer to the current challenges of schistosomiasis diagnosis and control in populations with lower parasite burden [63,64].

### Ultrasound abnormalities and chronic schistosomiasis

To our knowledge, this work is the first to systematically address the issue of structural abnormalities associated with schistosomiasis in the adult population of Mozambique. Studies in other endemic regions show that the prevalence of ultrasound findings in patients with schistosomiasis can be quite variable [41,65–67]. We found that 37.9% (95% CI 34.8%-41.2%) of adult individuals living in Chókwè HDSS had a structural urinary tract abnormality detected by ultrasound. *S*. *haematobium* detected by egg and/or molecular analysis was not a consistent predictor of ultrasound abnormality (p = 0.279, aOR 1.364 95% CI 0.778–2.392). Further analysis of ultrasound approach and findings will be detailed in an upcoming publication.

Contact with water from rivers, streams or lakes (p = 0.212, aOR 0.796 95% CI 0.556–1.139) and a history of schistosomiasis (p = 0.381, aOR 0.789 95% CI 0.465–1.340) were not found to be a risk factor for ultrasound abnormalities that might otherwise indicate a schistosomiasis related lesion. Serological diagnosis of schistosomiasis may have been helpful in our case, although previous studies note that serology alone is not a good predictor of ultrasound abnormalities, but it may be useful in combination with other findings such as blood in urine dipstick [68]. Our findings reinforce the value of ultrasound as an adjunct to diagnosis and screening in *S*. *haematobium* endemic areas. Ultrasound provides immediate data on disease-related changes associated with urinary schistosomiasis, but also has a remarkable correlation with other more invasive investigations such as cystoscopy [40,69]. A POCUS ultrasound approach should be considered as an important tool for routine screening of urinary tract lesions in schistosomiasis endemic areas, particularly in resource-limited settings where traditional diagnostic tools are less accessible.

### Schistosomiasis screening with anamnestic questionnaires

Screening questionnaires have been found to be an extremely useful method, as a first step in identifying communities with a high prevalence of schistosomiasis, and their use has been recommended by WHO in the Manual for Schistosomiasis since 1995 [70]. Over the years, the use of screening questionnaires has been tested and validated in several countries, confirming the good performance of this indirect method in identifying communities at high risk of *S*. *haematobium* infection [47,48]. However, in only one of these studies was the population studied in adulthood [71]. In a review of the use of urinary schistosomiasis screening questionnaires in sub-Saharan Africa, a total of 133880 children were screened using this method. The positive predictive value ranged from 31% to 92% and the negative predictive value from 75% to 100%, confirming the good performance of the questionnaires in screening for urinary schistosomiasis at the community level. On the other hand, their use in the individual screening for infection showed that reporting hematuria and a history of schistosomiasis were good indicators of infection. However, the use of individual questionnaires does not identify a high percentage of mild infections, which can be a problem in controlling morbidity [47]. With the introduction of mass chemotherapy as a control strategy, the high prevalence and morbidity associated with the disease decreased significantly [72]. We found that younger individuals (p = 0.011, aOR = 0.977, 95% CI 0.959–0.995) have a higher risk of being infected with *S*. *haematobium*. This finding seems logical, since younger individuals usually spend more time having recreational activities in rivers and lakes, and so, are more exposed to disease transmission.

Our results also confirmed that reported hematuria in the last month is a marker of *S*. *haematobium* infection (p = 0.002, aOR = 4.267, 95% CI 1.718–10.596), but did not find an association with a history of schistosomiasis or with any of the other health characteristics. Several studies have demonstrated the good correlation between reported hematuria and a high prevalence of *S*. *haematobium*, but in our case this correlation remains even in an adult population with low disease prevalence [47,73,74].

Another possible importance of screening questionnaires, is their use in identifying structural urinary tract morbidity associated with schistosomiasis. We found that people reporting lower abdominal pain in the last month (p = 0.017, aOR 1.599 95% CI 1.089–2.349) had higher chances of having an ultrasound abnormality. There was no significant association between reported hematuria (p = 0.511, aOR 1.340 95% CI 0.559–3.214), medical history including previous schistosomiasis infection (p = 0.381, aOR 0.789 95% CI 0.465–1.340) or contact with water from rivers, streams or lakes (p = 0.212, aOR 0.796 95% CI 0.556–1.139) with ultrasound abnormalities. This means that questions used to identify infected individuals in screening questionaries are not effective for identifying people with possible schistosomiasis associated urinary structural morbidity without a positive diagnostic test. Nevertheless, it may be worth further exploring the role of questions about bladder or lower abdominal pain as markers for ultrasound abnormalities. Another risk factor for an ultrasound abnormality was being born in Chókwè district (p = 0.007, aOR 0.608 95% CI 0.423–0.875), implying a possible longer or more intense exposure to disease transmission, as Chókwè district is home to one of the largest irrigation schemes in Mozambique and therefore has favorable conditions for disease transmission.

Community screening questionnaires have also been analyzed for *S*. *mansoni* infection, with positive predictive values ranging from 20% to 88% and negative predictive values ranging from 32% to 95%. The presence of blood in the stool and bloody diarrhea were the best diagnostic markers of infection [47,48,75]. Furst et al discuss the use of screening questionnaires in low endemicity areas and suggested that they have low sensitivity and specificity in identifying individuals infected with *S*. *mansoni* and geohelminths [76]. Our study confirms previous findings, as no association was found between any symptoms and *S*. *mansoni* infection in a low endemicity area. The only risk factor found for intestinal schistosomiasis was the use of water from rivers, streams or lakes for other activities (p = 0.017, aOR 2.452 95% CI 1.172–5.130).

### Urine dipsticks as a diagnostic marker

Like screening questionnaires, urine dipstick testing has been shown to be an effective, inexpensive and easy-to-use tool for identifying communities at high risk of infection [47,77]. However, their ability to detect infection is reduced in low prevalence areas, previously treated populations, or subgroups with mild infection. Urine dipsticks have a particularly low diagnostic accuracy for detecting ultra-light infections [78,79]. In our study, blood in urine is a marker of *S*. *haematobium* infection (p<0.001, aOR 3.193, 95% CI 1.834–5.560). In contrast, we found no association between blood in urine and ultrasound abnormalities (p = 0.348, aOR 1.237 95% 0.793–1.930). This could be partly explained by the type of the ultrasound abnormality, as in this analysis we do not distinguish between upper and lower urinary tract abnormalities, nor do we grade the severity of the lesions.

### Limitations and strengths

As the main strength of this study is the fact that it was the first to systematically evaluate schistosomiasis associated urinary tract abnormalities in an adult population of a rural district in Mozambique. Furthermore, it assessed *Schistosoma* spp. infection status using a combination of parasitological and molecular methods, thereby increasing the sensitivity and specificity of laboratory diagnosis of schistosomiasis, particularly in an area of low to moderate disease endemicity. In this cross-sectional study, we have simultaneously assessed schistosomiasis health related issues such as symptoms and medical history, water activities, performed several diagnostic methods and evaluated urinary tract abnormalities. However, there are several limitations to our study. First, the exact etiology of ultrasound abnormalities cannot be determined. In a schistosomiasis endemic area such as Chókwè district, we assume that the urinary tract structural lesions documented by ultrasound are probably a consequence of *Schistosoma* spp. infection, although the cross-sectional nature of the study design and the lack of other supporting laboratory data such as serology for schistosomiasis infection, limit the causal inference for the nature of the ultrasound abnormalities. Indeed, other risk factors for bladder lesions, such as smoking habits, were not addressed in the present study. Also, the presence of other diseases such as HIV and tuberculosis was assessed by questionnaires only and may be underestimated due to recall bias and social desirability. At last, the prevalence of kidney stones in Mozambique, a possible cause for ureter dilatation, is unknown. Second, the prevalence of other non-communicable diseases, such as diabetes and hypertension, other possible causes of urinary tract symptoms and urine dipstick abnormalities in the population studied, was also not addressed.

### Conclusions

The current study provides valuable insights into the morbidity associated with *S*. *haematobium*, characterizing for the first time the infection related urinary tract abnormalities in the adult population of Chókwè district and contributing to the understanding of the burden of the disease in Mozambique. Reported hematuria and blood in urine dipsticks are good infection markers, but do not appear to be important in urinary tract structural lesions identification.

## Supporting information

S1 Appendix Individual questionnaires in Portuguese and English. (PDF)

S2 Appendix Housing conditions questionnaires in Portuguese and English. (PDF)

S3 Appendix Ultrasound record sheet in Portuguese and English. (PDF)

S4 Appendix Laboratorial results for other intestinal parasites. (PDF)

S5 Appendix Association analysis for *S*. *haematobium* infection. (PDF)

S6 Appendix Association analysis for *S*. *mansoni* infection. (PDF)

S7 Appendix Association analysis for ultrasound abnormalities. (PDF)
