# Supplementary material for: Morbidity associated with schistosomiasis in adult population of Chókwè district, Mozambique
Source: PLoS Negl Trop Dis. 2024 Dec 16;18(12):e0012738. doi: 10.1371/journal.pntd.0012738 (PMC11684762; doi:10.1371/journal.pntd.0012738)
Supplement: S1 Appendix — (PDF) [file pntd.0012738.s001.pdf]

# Questionário individual

**Instituto de Higiene e Medicina Tropical da UNL/Faculdade de Medicina da UEM/Centro de Investigação e Treino em Saúde de Chókwè**

**1 Data:** ____________ **2 Código inquiridor:** ________ **3 Código:** ____________

## Dados sociodemográficos

**4 Data de nascimento:** ____________

**5 Há quanto tempo vive em Chókwè:**

- ☐ Menos de 1 ano
- ☐ Entre 1 a 2 anos
- ☐ Entre 3 a 5 anos
- ☐ Entre 6 a 10 anos
- ☐ Entre 11 a 20 anos
- ☐ Mais de 20 anos
- ☐ NS/NR

**6 Quantas pessoas vivem na sua casa:** ________

**7 Dessas pessoas, quantas têm menos de 15 anos:** ________

**8 Qual é o seu estado civil?**

- ☐ Solteiro(a)
- ☐ Casado(a)
- ☐ União marital
- ☐ Divorciado(a)
- ☐ Separado(a)
- ☐ Viúvo(a)
- ☐ NS/NR

**9 Qual é a sua ocupação:**

- ☐ Nenhuma
- ☐ Estudante
- ☐ Professor
- ☐ Agricultor
- ☐ Pastor
- ☐ Doméstica
- ☐ Carvoreiro/lenhador
- ☐ Comerciante
- ☐ Vendedor ambulante
- ☐ Guarda/segurança
- ☐ NS/NR
- ☐ Médico
- ☐ Enfermeiro
- ☐ Outra categ. saúde
- ☐ Agente Pol. Saúde
- ☐ Parteira tradicional
- ☐ Médico tradicional
- ☐ Militar ou polícia
- ☐ Carpinteiro
- ☐ Eletricista
- ☐ Serralheiro
- ☐ Pedreiro
- ☐ Religioso
- ☐ Reformado
- ☐ Empreg. doméstico
- ☐ Operário
- ☐ Cambista informal
- ☐ Incapacitado
- ☐ Voluntário
- ☐ Serviços da banca
- ☐ Motorista
- ☐ Gestor
- ☐ Trabalhador campo
- ☐ Cabeleireiro
- ☐ Jardineiro
- ☐ Técn. administrativo
- ☐ Desportista
- ☐ Artes e ofícios
- ☐ Biscateiro
- ☐ Servente
- ☐ Pintor
- ☐ Mecânico
- ☐ Recepcionista
- ☐ Alfaiate/modista
- ☐ Cozinheiro
- ☐ Músico
- ☐ Técnico informático
- ☐ Caçador
- ☐ Canalizador
- ☐ Promotor de eventos
- ☐ Outra

**10 Que nível de ensino completou:**

- ☐ Nenhum
- ☐ Alfabetização
- ☐ Primário EP1 (1 a 5)
- ☐ Primário EP2 (6 e 7)
- ☐ Secundário ESG1 (8 a 10)
- ☐ Secundário ESG2 (11 e 12)
- ☐ Técnico elementar
- ☐ Técnico básico
- ☐ Técnico médio
- ☐ Pós-graduado
- ☐ Superior
- ☐ NS/NR

**11 Em que sítio nasceu:**

- ☐ Distrito de Chókwè
- ☐ Outro distrito. Qual? ________
- ☐ Outra província. Qual? ________
- ☐ Outro país. Qual? ________
- ☐ NS/NR

**12 Sexo:**

- ☐ Masculino
- ☐ Feminino

## Dados clínicos

**13 Alguma vez teve sangue na urina?** ☐ Sim ☐ Não ☐ NS/NR

(Perguntar ao participante se alguma vez na vida urinou sangue ou urina de cor vermelha*)

**13.1 Se respondeu sim, desde há quanto tempo tem sangue na urina?**

(Perguntar desde há quanto tempo urina sangue ou urina de cor vermelha*)

- ☐ Menos de 1 ano
- ☐ Mais de 20 anos
- ☐ Entre 1 a 2 anos
- ☐ NS/NR
- ☐ Entre 3 a 5 anos
- ☐ Entre 6 a 10 anos
- ☐ Entre 11 a 20 anos

| As perguntas seguintes são relativas às queixas que teve no último mês: | Sim | Não | NS/NR |
|---|---|---|---|
| **14** No último mês, alguma vez teve sangue na urina?<br>(Perguntar apenas se respondeu sim à pergunta 13. Perguntar se urinou sangue ou teve urina de cor vermelha no último mês*) | ☐ | ☐ | ☐ |
| **15** No último mês, alguma vez doeu quando urinou?<br>(Perguntar se alguma vez doeu quando urinou no último mês*) | ☐ | ☐ | ☐ |
| **16** No último mês, alguma vez sentiu que não conseguia esvaziar a bexiga?<br>(Perguntar se alguma vez ficou com a sensação que queria urinar mais, mas não conseguia no último mês*) | ☐ | ☐ | ☐ |
| **17** No último mês, alguma vez teve dor de barriga?<br>(Perguntar se alguma vez teve dor de barriga no último mês. Classificar como sim se o participante disser que a dor de barriga é acima do umbigo, ou se disser que a dor é em toda a barriga.*) | ☐ | ☐ | ☐ |
| **18** No último mês, alguma vez teve dor na bexiga?<br>(Perguntar se alguma vez teve dor na barriga abaixo do umbigo no último mês. Classificar como sim se o participante disser que a dor de barriga é especificamente abaixo do umbigo. Se o participante referir apenas dor de barriga sem localizar, deve classificar como não.*) | ☐ | ☐ | ☐ |
| **19** No último mês, alguma vez teve diarreia?<br>(Perguntar se alguma vez teve diarreia no último mês. Apenas deve ser considerada diarreia, se o participante disser que teve pelo menos 3 dejeções líquidas num só dia.*) | ☐ | ☐ | ☐ |
| **20** No último mês, alguma vez teve sangue nas fezes?<br>(Perguntar se alguma vez teve sangue nas fezes no último mês.*) | ☐ | ☐ | ☐ |
| **21** No último mês, alguma vez teve parasitas/vermes nas fezes?<br>(Perguntar se alguma vezes viu parasitas ou outros vermes nas fezes no último mês.*) | ☐ | ☐ | ☐ |
| **22** No último mês, alguma vez teve febre?<br>(Perguntar se alguma vez teve febre no último mês. Classificar como sim desde que o participante o afirme, independentemente de ter usado termómetro para medir.*) | ☐ | ☐ | ☐ |

| As perguntas seguintes são relativas ás doenças que teve no passado: | Sim | Não | NS/NR |
|---|---|---|---|
| **23** Alguma vez teve Malária ou Paludismo? | ☐ | ☐ | ☐ |
| **24** Alguma vez teve Schistosomiase ou Bilharziose? | ☐ | ☐ | ☐ |
| **25** Alguma vez teve Filaríase ou Elefantíase? | ☐ | ☐ | ☐ |
| **26** Alguma vez teve parasitas ou vermes no intestino? | ☐ | ☐ | ☐ |
| **27** Alguma vez teve Oncocercose ou cegueira dos rios? | ☐ | ☐ | ☐ |
| **28** Alguma vez teve Tuberculose? | ☐ | ☐ | ☐ |
| **29** Alguma vez lhe disseram que tinha infeção VIH ou SIDA? | ☐ | ☐ | ☐ |

**30 Alguma vez fez tratamento para Schistosomiase ou Bilharziose?** ☐ Sim ☐ Não ☐ NS/NR

(Perguntar se alguma vez na vida recebeu tratamento específico para a Schistosomiase ou Bilharziose. Se o participante não souber ou não tiver a certeza, deve registar NS/NR. Usar uma amostra do medicamento para ajudar à identificação.*)

**30.1 Se respondeu sim, quando foi a última vez?**

☐ Menos de 1 ano ☐ Entre 1 a 2 anos ☐ Entre 3 a 5 anos ☐ Entre 6 a 10 anos ☐ Entre 11 a 20 anos
☐ Mais de 20 anos ☐ NS/NR

**31 Alguma vez fez tratamento para parasitas ou vermes intestinais?** ☐ Sim ☐ Não ☐ NS/NR
(Perguntar se alguma vez na vida recebeu tratamento específico para parasitas ou vermes intestinais. Se o participante não souber ou não tiver a certeza, deve registar NS/NR. Usar uma amostra do medicamento para ajudar à identificação.*)

**31.1 Se respondeu sim, quando foi a última vez?**

☐ Menos de 1 ano ☐ Entre 1 a 2 anos ☐ Entre 3 a 5 anos ☐ Entre 6 a 10 anos ☐ Entre 11 a 20 anos
☐ Mais de 20 anos ☐ NS/NR

**32 Está ou esteve menstruada nos últimos 2 dias?** ☐ Sim ☐ Não ☐ NS/NR
(Perguntar apenas aos participantes do sexo feminino. Perguntar se está menstruada no momento ou se terminou de menstruar há menos de 2 dias.*)

## Atividades relacionadas com a água

**As perguntas seguintes estão relacionadas com as atividades em que usa a água dos rios, riachos ou lagos:**
(Classificar como sim se o participante as realizou pelo menos 1 vez no último mês.*)

| | Sim | Não | NS/NR |
|---|---|---|---|
| **33** Costuma lavar a roupa com água do rio, riacho ou lago? | ☐ | ☐ | ☐ |
| **34** Costuma lavar a loiça com água do rio, riacho ou lago? | ☐ | ☐ | ☐ |
| **35** Costuma tomar banho no rio, riacho ou lago? | ☐ | ☐ | ☐ |
| **36** Costuma dar banho ás crianças com água do rio, riacho ou lago? | ☐ | ☐ | ☐ |
| **37** Costuma nadar nos rios, riachos ou lagos? | ☐ | ☐ | ☐ |
| **38** Costuma atravessar rios, riachos ou lagos? | ☐ | ☐ | ☐ |
| **39** Costuma cozinhar com água do rio, riacho ou lago? | ☐ | ☐ | ☐ |
| **40** Costuma pescar com redes nos rios, riachos ou lagos? | ☐ | ☐ | ☐ |
| **41** Costuma pescar com cana nos rios, riachos ou lagos? | ☐ | ☐ | ☐ |
| **42** Utiliza água dos rios, riachos ou lagos para agricultura? | ☐ | ☐ | ☐ |
| **43** Utiliza água dos rios, riachos ou lagos para práticas religiosas? | ☐ | ☐ | ☐ |
| **44** Utiliza água dos rios, riachos ou lagos para outras atividades? | ☐ | ☐ | ☐ |

**As perguntas seguintes estão relacionados com o uso de sabão:**
(Classificar como sim se o participante as realiza a maioria das vezes [<50%].*)

| | Sim | Não | NS/NR |
|---|---|---|---|
| **45** Costuma usar sabão para lavar a roupa? | ☐ | ☐ | ☐ |
| **46** Costuma usar sabão para lavar a loiça? | ☐ | ☐ | ☐ |
| **47** Costuma usar sabão para lavar as mãos? | ☐ | ☐ | ☐ |
| **48** Costuma usar sabão para tomar banho? | ☐ | ☐ | ☐ |

## Observações

* Instruções explicativas sobre o significado e modo de colocar a questão ao entrevistado

NS/NR - Não Sabe ou Não Responde

## Individual questionnaire

**Institute of Hygiene and Tropical Medicine of NOVA University/Faculty of Medicine - Eduardo Mondale University/Chókwè Health Research and Training Center**

1 **Date:** ____________ 2 **Inquiring code:** ____________ 3 **Code:** ____________

### Sociodemographic data

4 **Date of birth:** ____________

6 **How many people live in your house:** ____________

5 **How long have you lived in Chókwè:**

- ☐ Less than 1 year
- ☐ Between 1 to 2 years
- ☐ Between 3 to 5 years
- ☐ Between 6 to 10 years
- ☐ Between 11 and 20 years
- ☐ More than 20 years
- ☐ DK/RA

7 **Of these people, how many are under 15:** ____________

8 **What is your marital status:**

- ☐ Single
- ☐ Married
- ☐ Cohabiting
- ☐ Divorced
- ☐ Separated
- ☐ Widower
- ☐ DK/RA

9 **What is your profession:**

- ☐ None
- ☐ Student
- ☐ Teacher
- ☐ Farmer
- ☐ Shepperd
- ☐ Domestic
- ☐ Carver/woodcutter
- ☐ Merchant
- ☐ Street informal vendor
- ☐ Guard/security
- ☐ DK/RA
- ☐ Doctor
- ☐ Nurse
- ☐ Other health worker
- ☐ Comunity health worker
- ☐ Traditional midwife
- ☐ Traditional doctor
- ☐ Military or police
- ☐ Carpenter
- ☐ Electrician
- ☐ Locksmith
- ☐ Mason
- ☐ Priest
- ☐ Retired
- ☐ Housekeeper
- ☐ Factory worker
- ☐ Money changer
- ☐ Person with disability
- ☐ Voluntary worker
- ☐ Banking worker
- ☐ Driver
- ☐ Manager
- ☐ Rural worker
- ☐ Hairdresser
- ☐ Gardener
- ☐ Technical staff
- ☐ Sportsman
- ☐ Arts and crafts
- ☐ Handyman
- ☐ Servant
- ☐ Painter
- ☐ Mechanic
- ☐ Receptionist
- ☐ Tailor/dresser
- ☐ Chef
- ☐ Musician
- ☐ IT technician
- ☐ Hunter
- ☐ Plumber
- ☐ Event promoter
- ☐ Other

10 **What level of education have you completed:**

- ☐ None
- ☐ Literacy
- ☐ Primary EP1 (1 to 5)
- ☐ Primary EP2 (6 and 7)
- ☐ Secondary ESG1 (8 to 10)
- ☐ Secondary ESG2 (11 and 12)
- ☐ Elementary technician
- ☐ Basic technician
- ☐ Medium Technician
- ☐ Postgraduate
- ☐ Higher education
- ☐ DK/RA

11 **Where were you born:**

- ☐ Chókwè district
- ☐ Another district. Which? ____________
- ☐ Another province. Which? ____________
- ☐ Another country. Which? ____________
- ☐ DK/RA

12 **Sex:**

- ☐ Male
- ☐ Female

### Clinical data

13 **Have you ever had blood in your urine?** ☐ Yes ☐ No ☐ DK/RA

(Ask the participants if he or she has ever urinated blood or had red urine*)

13.1 **If yes, for how long have you had blood in your urine?**

(Ask for how long he or she has been urinating blood or had red urine*)

- ☐ Less than 1 year
- ☐ Between 1 to 2 years
- ☐ Between 3 to 5 years
- ☐ Between 6 to 10 years
- ☐ Between 6 to 10 years
- ☐ Between 11 and 20 years
- ☐ More then 20 years
- ☐ DK/RA

| **The following questions are related to the complaints you have had in the last month:** | Yes | No | DK/RA |
|---|---|---|---|
| **14** In the last month, have you ever had blood in your urine?<br>(Just ask if answered yes to question 13. Ask if he or she has urinated blood or had red urine in the last month*) | ☐ | ☐ | ☐ |
| **15** In the last month, has it ever hurt when you urinated?<br>(Ask if he or she has ever felt pain when urinating in the last month*) | ☐ | ☐ | ☐ |
| **16** In the last month, have you ever felt like you couldn't empty your bladder?<br>(Ask if he or she has ever felt like wanting to urinate more, but couldn't do it in the last month*) | ☐ | ☐ | ☐ |
| **17** In the last month, have you ever had abdominal pain?<br>(Ask if he or she has ever had abdominal pain in the last month. Classify as yes if he or she says that the abdominal pain is above the belly button or if the pain is all over the belly.*) | ☐ | ☐ | ☐ |
| **18** In the last month, have you ever had lower abdominal pain?<br>(Ask if he or she has ever had abdominal pain below the belly button in the last month. Classify as yes if he or she says the abdominal pain is specifically bellow the belly button. If the participant only reports abdominal pain without localizing, classify as no.*) | ☐ | ☐ | ☐ |
| **19** In the last month, have you ever had diarrhea?<br>(Ask if you he or she had diarrhea in the last month. It should only be considered diarrhea, if the participant says they had at least 3 liquid bowel movements in a single day.*) | ☐ | ☐ | ☐ |
| **20** In the last month, have you ever had blood in your stools?<br>(Ask if you he or she ever had blood in the stools in the last month.*) | ☐ | ☐ | ☐ |
| **21** In the last month, have you ever had parasites/worms in your stools?<br>(Ask if he or she has ever seen parasites or other worms in the stools in the last month.*) | ☐ | ☐ | ☐ |
| **22** In the last month, have you ever had a fever?<br>(Ask if you he or she ever had a fever in the last month. Classify as yes as long as the participant says so, regardless of whether he or she used a thermometer to measure.*) | ☐ | ☐ | ☐ |

| **The following questions are related to illnesses you might have had in the past:** | Yes | No | DK/RA |
|---|---|---|---|
| **23** Have you ever had Malaria? | ☐ | ☐ | ☐ |
| **24** Have you ever had Schistosomiasis or Bilharzia? | ☐ | ☐ | ☐ |
| **25** Have you ever had Filariasis or Elephantiasis? | ☐ | ☐ | ☐ |
| **26** Have you ever had parasites or worms in your intestines? | ☐ | ☐ | ☐ |
| **27** Have you ever had Onchocerciasis or river blindness? | ☐ | ☐ | ☐ |
| **28** Have you ever had Tuberculosis? | ☐ | ☐ | ☐ |
| **29** Have you ever been told that you had HIV infection or AIDS? | ☐ | ☐ | ☐ |

**30 Have you ever been treated for Schistosomiasis or Bilharzia?** ☐ Yes ☐ No ☐ DK/RA

(Ask if he or she has ever received specific treatment for Schistosomiasis or Bilharzia in life. If the participant does not know or is not sure, classify as DK/NA Use a medicine sample to help with identification.*)

**30.1 If you answered yes, when was the last time?**

| | | | |
|---|---|---|---|
| ☐ Less than 1 year | ☐ Between 3 to 5 years | ☐ Between 6 to 10 years | ☐ More then 20 years |
| ☐ Between 1 to 2 years | ☐ Between 6 to 10 years | ☐ Between 11 and 20 years | ☐ DK/RA |

**31 Have you ever been treated for parasites or intestinal worms?** ☐ Yes ☐ No ☐ DK/RA

(Ask if he or she has ever received specific treatment for parasites or intestinal in life. If the participant does not know or is not sure, classify as DK/NA. Use a medicine smple to help with identification.*)

**31.1 If you answered yes, when was the last time?**

☐ Less than 1 year
☐ Between 1 to 2 years
☐ Between 3 to 5 years
☐ Between 6 to 10 years
☐ Between 6 to 10 years
☐ Between 11 and 20 years
☐ More then 20 years
☐ DK/RA

**32 Are you or have you been menstruating in the last 2 days?** ☐ Yes ☐ No ☐ DK/RA

(Only ask female participants. Ask if she is menstruating at the moment or if finished menstruating less than 2 days ago.*)

## Water activities

**The following questions are related to activities in which you use water from rivers, streams or lakes:**

(Classify as yes if the participant performed them at least once in the last month.*)

| | | Yes | No | DK/RA |
|---|---|---|---|---|
| **33** | Do you usually wash your clothes with rivers, streams or lake water? | ☐ | ☐ | ☐ |
| **34** | Do you usually wash dishes with rivers, streams or lake water? | ☐ | ☐ | ☐ |
| **35** | Do you usually bathe in the river, stream or lake? | ☐ | ☐ | ☐ |
| **36** | Do you usually bathe your children with rivers, streams or lake water? | ☐ | ☐ | ☐ |
| **37** | Do you usually swim in rivers, streams or lakes? | ☐ | ☐ | ☐ |
| **38** | Do you often cross rivers, streams or lakes? | ☐ | ☐ | ☐ |
| **39** | Do you usually cook with rivers, streams or lake water? | ☐ | ☐ | ☐ |
| **40** | Do you usually fish with nets in rivers, streams or lakes? | ☐ | ☐ | ☐ |
| **41** | Do you usually fish with a rod in rivers, streams or lakes? | ☐ | ☐ | ☐ |
| **42** | Do you use water from rivers, streams or lakes for agriculture? | ☐ | ☐ | ☐ |
| **43** | Do you use water from rivers, streams or lakes for religious practices? | ☐ | ☐ | ☐ |
| **44** | Do you use water from rivers, streams or lakes for other activities? | ☐ | ☐ | ☐ |

**The following questions are related to soap use:**

(Classify as yes if the participant performs them most of the time [> 50%]*)

| | | Yes | No | DK/RA |
|---|---|---|---|---|
| **45** | Do you usually use soap to wash your clothes? | ☐ | ☐ | ☐ |
| **46** | Do you usually use soap to wash the dishes? | ☐ | ☐ | ☐ |
| **47** | Do you usually use soap to wash your hands? | ☐ | ☐ | ☐ |
| **48** | Do you usually use soap to bathe? | ☐ | ☐ | ☐ |

## Comments

* Explanatory instructions on the meaning and way of asking the question to the interviewee

DK/RA - Don't Know or Refuse to answer
