# Supplementary material for: Morbidity associated with schistosomiasis in adult population of Chókwè district, Mozambique
Source: PLoS Negl Trop Dis. 2024 Dec 16;18(12):e0012738. doi: 10.1371/journal.pntd.0012738 (PMC11684762; doi:10.1371/journal.pntd.0012738)
Supplement: S2 Appendix — (PDF) [file pntd.0012738.s002.pdf]

Questionário de condições habitacionais

Instituto de Higiene e Medicina Tropical da UNL/Faculdade de Medicina da UEM/Centro de Investigação e Treino em Saúde de Chókwè

1 Data: ______________ **2 Código inquiridor:** ________ **3 ID_agregado:** ___________ **4 Código:** ______________

Este questionário deve ser aplicado apenas a um dos membros do agregado familiar

5 Qual a fonte da água que usa na sua casa?

- ☐ Água canalizada dentro de casa
- ☐ Água canalizada fora de casa/quintal
- ☐ Água canalizada torneira pública/fontanário
- ☐ Água canalizada na casa do vizinho
- ☐ Água do poço ou furo com bomba manual
- ☐ Água do poço sem bomba manual
- ☐ Água do poço não protegido
- ☐ Água da chuva
- ☐ Água do rio/riacho/lago
- ☐ Água engarrafada/mineral
- ☐ Outra ________________
- ☐ NS/NR

5.1 Onde está localizada a fonte da água?

- ☐ Dentro de casa
- ☐ Centro do quintal
- ☐ Fora do quintal/casa
- ☐ Na casa do vizinho
- ☐ Outra ______________
- ☐ NS/NR

6 A sua casa tem casa de banho? ☐ Sim ☐ Não ☐ NS/NR

6.1 Se respondeu sim, que tipo de casa de banho tem?

- ☐ Retrete com autoclismo
- ☐ Retrete sem autoclismo
- ☐ Latrina melhorada
- ☐ NS/NR
- ☐ Latrina tradicional melhorada
- ☐ Latrina não melhorada
- ☐ Outra ________________

7 A sua casa tem eletricidade?

☐ Sim ☐ Não ☐ NS/NR

8 A sua casa tem cozinha?

☐ Sim ☐ Não ☐ NS/NR

9 Que combustível usa para cozinhar?

☐ Lenha ☐ Eletricidade ☐ Gás natural ☐ Petróleo ☐ Carvão ☐ Outra ________ ☐ NS/NR

NS/NR - Não Sabe ou Não Responde

Housing conditions questionnaire

Institute of Hygiene and Tropical Medicine of NOVA University/Faculty of Medicine - Eduardo Mondale University/Chókwè Health Research and Training Center

1 **Date:** ______________ 2 **Inquiring code:** ___________ 3 **House_ID:** ________________ 4 **Code:** ________________

This questionnaire must only be applied to one member of the household

5 What is the source of the water you use in your house?

- [ ] Piped water inside the house
- [ ] Piped water outside the house/yard
- [ ] Piped water public tap/standpipe
- [ ] Piped water in the neighbor's house
- [ ] Water from well or borehole with hand pump
- [ ] Well water without hand pump
- [ ] Unprotected well water
- [ ] Rain water
- [ ] River/stream/lake water
- [ ] Bottled/mineral water
- [ ] Other ________________
- [ ] DK/RA

5.1 Where is the water source located?

- [ ] Inside home
- [ ] Center of the yard
- [ ] Outside the yard/house
- [ ] At the neighbor's house
- [ ] Other ______________
- [ ] DK/RA

6 Does your house have a bathroom?

- [ ] Yes
- [ ] No
- [ ] DK/RA

6.1 If you answered yes, what kind of bathroom do you have?

- [ ] Toilet with flush toilet
- [ ] Toilet without flushing
- [ ] Improved latrine
- [ ] DK/RA
- [ ] Improved traditional latrine
- [ ] Basic latrine
- [ ] Other ______________

7 Does your house have electricity?

- [ ] Yes
- [ ] No
- [ ] DK/RA

8 Does your house have a kitchen?

- [ ] Yes
- [ ] No
- [ ] DK/RA

9 What fuel do you use to cook?

- [ ] Firewood
- [ ] Electricity
- [ ] Natural gas
- [ ] Petroleum
- [ ] Coal
- [ ] Other ________
- [ ] DK/RA

DK/RA - Don`t Know or Refuse to Answer
