# Supplementary material for: Morbidity associated with schistosomiasis in adult population of Chókwè district, Mozambique
Source: PLoS Negl Trop Dis. 2024 Dec 16;18(12):e0012738. doi: 10.1371/journal.pntd.0012738 (PMC11684762; doi:10.1371/journal.pntd.0012738)
Supplement: S3 Appendix — (PDF) [file pntd.0012738.s003.pdf]

Formulário de registo de dados ecográficos

**Altura:** ______ cm	**Peso:** ______ Kg	**Data:** ____________	**Código:** __________

Bexiga (avaliação deve ser realizada <u>prévia</u> ao esvaziamento vesical)

Volume	Diâmetro DE: _______ cm	Diâmetro CC: _______ cm	Diâmetro AP: _______ cm	**Score**
Conteúdo	Focos ecogénicos no interior do lúmen vesical (Não = 0; Sim =1)			
Parede	Sem espessamento da parede (<5mm) e sem lesões intraluminais = 0<br>Irregularidade ou espessamento da parede entre 5-7mm sem lesões intraluminais = 1<br>Irregularidade ou espessamento da parede entre 8-9mm sem lesões intraluminais = 2<br>Irregularidade ou espessamento da parede ≥10mm ou qualquer pólipo, massa ou tumor = 3			
	Score trato urinário inferior			

**Ureteres** (avaliação deve ser realizada <u>após</u> esvaziamento vesical)<br>[classificar de acordo com a lesão mais grave observada]		**Score**
Avaliar os dois	Ureter não visualizado ou com diâmetro ≤ 3mm = 0<br>Diâmetro do ureter >3mm em qualquer extensão (proximal, distal ou todo comprimento) = 1	
**Rins** (avaliação deve ser realizada <u>após</u> esvaziamento vesical)<br>[classificar de acordo com a lesão mais grave observada]		**Score**
Avaliar os dois	Pelve renal não visualizada ou < 2mm = 0<br>Pelve renal entre 2mm e 5mm = 1<br>Pelve renal entre 6mm e 10mm = 2<br>Pelve renal ≥ 11mm = 3	
	Score trato urinário superior	
	**Score ecográfico final**	

Notas

DE (direito-esquerdo); CC (cranio-caudal); AP (antero-posterior)

Ultrasound record sheet

Height: ______ cm	Weight: ______ Kg	Date: ____________	Code: ____________

**Bladder** (evaluation must be done <u>before</u> voiding)				
Volume	RL length: _________ cm	CC length: _________ cm	AP length: _________ cm	**Score**
Content	Echogenic foci inside bladder lumen (No = 0; Yes = 1)			
Wall	No wall thickening (<5mm) without intraluminal lesions = 0<br>Irregularity or thickening of the wall between 5-7mm without intraluminal lesions = 1<br>Irregularity or thickening of the wall between 8-9mm without intraluminal lesions = 2<br>Irregularity or thickening of the wall ≥10mm or any polyps, masses or tumors = 3			
	Lower urinary tract score			
**Ureters** (evaluation must be done <u>after</u> voiding)<br>[Score accordingly to the most severe lesion seen]				**Score**
Evaluate both	Ureter not shown or with a diameter ≤ 3mm = 0<br>Dilation of the ureter (>3mm) in any extension (proximal, distal or full extent) = 1			
**Kidneys** (evaluation must be done <u>after</u> voiding)<br>[Score accordingly to the most severe lesion seen]				**Score**
Evaluate both	Renal pelvis fissure not shown or < 2mm = 0<br>Renal pelvis fissure between 2mm and 5mm = 1<br>Renal pelvis fissure between 6mm and 10mm = 2<br>Renal pelvis fissure ≥ 11mm = 3			
	Upper urinary tract score			
	**Final ultrasound score**			

Notes

RL (right-left); CC (craniocaudal); AP (anterior-posterior)
