# Supplementary material for: Morbidity associated with schistosomiasis in adult population of Chókwè district, Mozambique
Source: PLoS Negl Trop Dis. 2024 Dec 16;18(12):e0012738. doi: 10.1371/journal.pntd.0012738 (PMC11684762; doi:10.1371/journal.pntd.0012738)
Supplement: S4 Appendix — (PDF) [file pntd.0012738.s004.pdf]

Laboratorial results for other intestinal parasites

**Parasite**	**Parasitological diagnosis** n (%)
Trichuris trichiura	2/922 (0.2)
Ascaris lumbricoides	66/922 (7.2)
Hookworm	6/922 (0.7)
