# Supplementary material for: Morbidity associated with schistosomiasis in adult population of Chókwè district, Mozambique
Source: PLoS Negl Trop Dis. 2024 Dec 16;18(12):e0012738. doi: 10.1371/journal.pntd.0012738 (PMC11684762; doi:10.1371/journal.pntd.0012738)
Supplement: S5 Appendix — (PDF) [file pntd.0012738.s005.pdf]

## Association analysis for *S. haematobium* infection

| Variable | | *Schistosoma haematobium* infection | |
|---|---|---|---|
| | | **p value** | **Crude OR (95% CI)** |
| Sex (ref: male) | | 0.651 | 1.105 (0.716-1.708) |
| Age | | 0.003 | 0.976 (0.964-0.987) |
| Marital status | Single | 0.501 | - |
| | Married or cohabiting | | - |
| | Divorced or separated | | - |
| | Widower | | - |
| Education | None | 0.055 | - |
| | Primary | | - |
| | Secondary | | - |
| | Higher education and post-graduation | | - |
| Occupation | Unemployed or retired | 0.009 | - |
| | Student | | 0.617 (0.340-1.120) |
| | Agriculture and fishing | | 0.780 (0.488-1.245) |
| | Industry and construction | | 0.856 (0.374-1.959) |
| | Trade and services | | 0.236 (0.096-0.575) |
| | Others | | - |
| Place of birth (ref: Chókwè district) | | 0.204 | - |
| Time of residency (ref: <=20 years) | | <0.001 | 0.397 (0.268-0.587) |
| Number of people living in the same house | | 0.880 | - |
| Number of people in the same house less then 15 years old | | 0.004 | 1.064 (0.971-1.166) |
| Water source (ref: piped water) | | 0.003 | 2.125 (1.273-3.547) |
| Water source location | Inside home | 0.013 | - |
| | Inside the backyard | | 1.523 (0.356-6.516) |
| | Outside the house or yard | | 3.040 (0.644-14.362) |
| | At the neighbor's house | | 2.747 (0.602-12.548) |
| | Other | | 3.491 (0.737-16.539) |
| Bathroom/Sanitation facilities | No | 0.060 | - |
| | Toilet | | - |
| | Improved latrine | | - |
| | Basic latrine | | - |
| | Other | | - |
| Electricity (ref: no) | | <0.001 | 0.458 (0.299-0.700) |
| Kitchen (ref: no) | | 0.555 | - |
| Cooking fuel | Firewood | 0.008 | - |
| | Electricity | | 4.418 (1.407-13.875) |
| | Coal | | 0.724 (0.479-1.092) |
| | Other | | - |
| Hematuria, any time in live (ref: no) | | 0.004 | 1.803 (1.205-2.698) |
| Time since last hematuria episode | | 0.200 | |
| Hematuria, last month (ref: no) | | <0.001 | 6.944 (3.397-14.198) |
| Dysuria, last month (ref: no) | | 0.273 | - |
| Difficulty emptying the bladder, last month (ref: no) | | 0.278 | - |
| Abdominal pain, last month (ref: no) | | 0.356 | - |
| Lower abdominal pain, last month (ref: no) | | 0.157 | - |
| Diarrhea, last month (ref: no) | | 0.404 | - |
| Blood in stool, last month (ref: no) | | 0.238 | - |
| Worms or parasites in stool, last month (ref: no) | | 0.274* | - |
| Fever, last month (ref: no) | | 0.543 | - |
| Malaria, anytime in the past (ref: no) | | 0.038 | 2.391 (1.025-5.578) |
| Schistosomiasis, anytime in the past (ref: no) | | 0.122 | - |
| Filariasis, anytime in the past (ref: no) | | 0.999* | - |
| Worms or intestinal parasites, anytime in the past (ref: no) | | 0.548 | - |
| Onchocerciasis, anytime in the past (ref: no) | | No data | - |
| Tuberculosis, anytime in the past (ref: no) | | 0.642 | - |
| HIV infection, anytime in the past (ref: no) | | 0.708 | - |
| Schistosomiasis treatment, anytime in the past (ref: no) | | 0.308 | - |
| Time since last Schistosomiasis treatment (ref: ≤ 20 years) | | 0.382 | - |
| Intestinal parasites treatment, anytime in the past (ref: no) | | 0.642 | - |
| Time since last intestinal parasites treatment (ref: ≤ 20 years) | | 0.715* | - |
| To do the laundry with water from rivers, streams or lakes (ref: no) | | 0.003 | 2.265 (1.310-3.914) |
| To wash dishes with water from rivers, streams or lakes (ref: no) | | 0.220 | - |
| To wash yourself with water from rivers, streams or lakes (ref: no) | | 0.018 | 1.719 (1.091-2.711) |
| To wash the children with water from rivers, streams or lakes (ref: no) | | 0.579* | - |
| To swim in water from rivers, streams or lakes (ref: no) | | 0.011* | 2.754 (1.310-5.791) |

| | | |
|---|---|---|
| To cross rivers, streams or lakes (ref: no) | 0.040 | 1.520 (1.016-2.275) |
| To cook with water from rivers, streams or lakes (ref: no) | 0.887 | - |
| To fish with a net in rivers, streams or lakes (ref: no) | 0.999* | - |
| To fish with a hook rivers, streams or lakes (ref: no) | 0.037 | 2.876 (1.017-8.136) |
| To use water from rivers, streams or lakes for agriculture activities (ref: no) | 0.012 | 1.662 (1.115-2.576) |
| To use water from rivers, streams or lakes for religious activities (ref: no) | 0.224* | - |
| To use water from rivers, streams or lakes for other activities (ref: no) | 0.022 | 1.819 (1.085-3.049) |
| To use soap to do the laundry (ref: no) | 0.713* | - |
| To use soap to wash dishes (ref: no) | 0.807* | - |
| To use soap to wash hands (ref: no) | 0.248 | - |
| To use soap to wash yourself (ref: no) | 0.400* | - |
| Blood in urine dipstick (ref: no) | <0.001 | 4.629 (3.065-6.992) |
| Proteinuria in urine dipstick (ref: no) | <0.001 | 2.911 (1.865-4.543) |
| Leukocytes in urine dipstick (ref: no) | 0.009 | 2.311 (1.210-4.414) |
| Nitrites in urine dipstick (ref: no) | 0.031* | 2.549 (1.175-5.527) |
| *Schistosoma mansoni* infection (ref: no) | 0.176 | - |
| Ultrasound abnormality (ref: no) | 0.005 | 1.823 (1.197-2.777) |
| *Ascaris lumbricoides* (ref: no) | 0.542 | - |
| Hookworm (ref: no) | 0.517* | - |
| *Trichuris trichiura* (ref: no) | 0.215* | - |

ref, reference class

*Bivariate analysis using Fisher`s exact test
