# Supplementary material for: Morbidity associated with schistosomiasis in adult population of Chókwè district, Mozambique
Source: PLoS Negl Trop Dis. 2024 Dec 16;18(12):e0012738. doi: 10.1371/journal.pntd.0012738 (PMC11684762; doi:10.1371/journal.pntd.0012738)
Supplement: S6 Appendix — (PDF) [file pntd.0012738.s006.pdf]

Association analysis for *S. mansoni* infection

Variable		*Schistosoma mansoni* infection	
		p value	Crude OR (95% CI)
Sex (ref: male)		0.035	2.234 (1.038-4.807)
Age		0.357	1.003 (0.989-1.018)
Marital status	Single	0.125	-
	Married or cohabiting		-
	Divorced or separated		-
	Widower		-
Education	None	0.131	-
	Primary		-
	Secondary		-
	Higher education and post-graduation		-
Occupation	Unemployed or retired	0.085	-
	Student		-
	Agriculture and fishing		-
	Industry and construction		-
	Trade and services		-
	Others		-
Place of birth (ref: Chókwè district)		0.253	-
Time of residency (ref: <=20 years)		0.408	-
Number of people living in the same house		0.660	-
Number of people in the same house less then 15 years old		0.143	-
Water source (ref: piped water)		0.648	-
Water source location	Inside home	0.404	-
	Inside the backyard		-
	Outside the house or yard		-
	At the neighbor's house		-
	Other		-
Bathroom/Sanitation facilities	No	0.055	-
	Toilet		-
	Improved latrine		-
	Basic latrine		-
	Other		-
Electricity (ref: no)		0.332	-
Kitchen (ref: no)		0.019	0.497 (0.275-0.899)
Cooking fuel	Firewood	0.768	-
	Electricity		-
	Coal		-
	Other		-
Hematuria, any time in live (ref: no)		0.686	-
Time since last hematuria episode		0.381*	-
Hematuria, last month (ref: no)		0.999*	-
Dysuria, last month (ref: no)		0.419	-
Difficulty emptying the bladder, last month (ref: no)		0.137	-
Abdominal pain, last month (ref: no)		0.428	-
Lower abdominal pain, last month (ref: no)		0.241	-
Diarrhea, last month (ref: no)		0.758	-
Blood in stool, last month (ref: no)		0.797*	-
Worms or parasites in stool, last month (ref: no)		0.428*	-
Fever, last month (ref: no)		0.946	-
Malaria, anytime in the past (ref: no)		0.536	-
Schistosomiasis, anytime in the past (ref: no)		0.328	-
Filariasis, anytime in the past (ref: no)		0.999*	-
Worms or intestinal parasites, anytime in the past (ref: no)		0.157	-
Onchocerciasis, anytime in the past (ref: no)		No data	-
Tuberculosis, anytime in the past (ref: no)		0.892	-
HIV infection, anytime in the past (ref: no)		0.730	-
Schistosomiasis treatment, anytime in the past (ref: no)		0.110	-
Time since last Schistosomiasis treatment (ref: ≤ 20 years)		0.999*	-
Intestinal parasites treatment, anytime in the past (ref: no)		0.303*	-
Time since last intestinal parasites treatment (ref: ≤ 20 years)		0.999*	-
To do the laundry with water from rivers, streams or lakes (ref: no)		0.004*	3.025 (1.490-6.141)
To wash dishes with water from rivers, streams or lakes (ref: no)		0.145*	-
To wash yourself with water from rivers, streams or lakes (ref: no)		0.031	1.968 (1.055-13.672)
To wash the children with water from rivers, streams or lakes (ref: no)		0.034*	3.180 (1.175-8.609)
To swim in water from rivers, streams or lakes (ref: no)		0.999*	-

To cross rivers, streams or lakes (ref: no)	0.188	-
To cook with water from rivers, streams or lakes (ref: no)	0.040*	2.483 (1.067-5.781)
To fish with a net in rivers, streams or lakes (ref: no)	0.340*	-
To fish with a hook in rivers, streams or lakes (ref: no)	0.999*	-
To use water from rivers, streams or lakes for agriculture activities (ref: no)	0.215	-
To use water from rivers, streams or lakes for religious activities (ref: no)	0.999*	-
To use water from rivers, streams or lakes for other activities (ref: no)	<0.001	2.921 (1.530-5.577)
To use soap to do the laundry (ref: no)	0.618*	-
To use soap to wash dishes (ref: no)	0.500*	-
To use soap to wash hands (ref: no)	0.751	-
To use soap to wash yourself (ref: no)	0.999*	-
Blood in urine dipstick (ref: no)	0.730	1.129 (0.567-2.244)
Proteinuria in urine dipstick (ref: no)	0.256	-
Leukocytes in urine dipstick (ref: no)	0.766*	-
Nitrites in urine dipstick (ref: no)	0.016*	3.451 (1.372-8.679)
Schistosoma haematobium infection (ref: no)	0.176	-
Ultrasound abnormality (ref: no)	0.908	-
Ascaris lumbricoides (ref: no)	0.420*	-
Hookworm (ref: no)	0.300	-
Trichuris trichiura (ref: no)	0.999*	-

ref, reference class

*Bivariate analysis using Fisher`s exact test
