# Supplementary material for: Morbidity associated with schistosomiasis in adult population of Chókwè district, Mozambique
Source: PLoS Negl Trop Dis. 2024 Dec 16;18(12):e0012738. doi: 10.1371/journal.pntd.0012738 (PMC11684762; doi:10.1371/journal.pntd.0012738)
Supplement: S7 Appendix — (PDF) [file pntd.0012738.s007.pdf]

Association for ultrasound abnormalities

Variable		Ultrasound abnormality	
		p value	Crude OR (95% CI)
Sex (ref: male)		0.171	1.235 (0.913-1.672)
Age		0.184	1.000 (0.993-1.007)
Marital status	Single	0.655	-
	Married or cohabiting		-
	Divorced or separated		-
	Widower		-
Education	None	0.218	-
	Primary		-
	Secondary		-
	Higher education and post-graduation		-
Occupation	Unemployed or retired	0.522	-
	Student		-
	Agriculture and fishing		-
	Industry and construction		-
	Trade and services		-
	Others		-
Place of birth (ref: Chókwè district)		0.026	0.734 (0.558-0.965)
Time of residency (ref: <=20 years)		0.746	-
Number of people living in the same house		0.944	-
Number of people in the same house less then 15 years old		0.883	-
Water source (ref: piped water)		0.107	-
Water source location	Inside home	0.124	-
	Inside the backyard		-
	Outside the house or yard		-
	At the neighbor's house		-
	Other		-
Bathroom/Sanitation facilities	No	0.197	-
	Toilet		-
	Improved latrine		-
	Basic latrine		-
	Other		-
Electricity (ref: no)		0.166	0.787 (0.561-1.105)
Kitchen (ref: no)		0.718	0.942 (0.679-1.305)
Cooking fuel	Firewood	0.024	-
	Electricity		1.101 (0.292-4.147)
	Coal		0.658 (0.497-0.870)
	Other		-
Hematuria, any time in live (ref: no)		0.170	-
Time since last hematuria episode		0.499	-
Hematuria, last month (ref: no)		0.001	3.088 (1.593-6.261)
Dysuria, last month (ref: no)		<0.001	1.772 (1.331-2.359)
Difficulty emptying the bladder, last month (ref: no)		0.011	1.475 (1.094-1.990)
Abdominal pain, last month (ref: no)		0.931	-
Lower abdominal pain, last month (ref: no)		0.001	1.577 (1.196-2.079)
Diarrhea, last month (ref: no)		0.443	-
Blood in stool, last month (ref: no)		0.016	1.794 (1.111-2.895)
Worms or parasites in stool, last month (ref: no)		0.696	-
Fever, last month (ref: no)		0.677	-
Malaria, anytime in the past (ref: no)		0.154	-
Schistosomiasis, anytime in the past (ref: no)		0.138	1.264 (0.927-1.723)
Filariasis, anytime in the past (ref: no)		0.529*	-
Worms or intestinal parasites, anytime in the past (ref: no)		0.759	-
Onchocerciasis, anytime in the past (ref: no)		No data	-
Tuberculosis, anytime in the past (ref: no)		0.066	-
HIV infection, anytime in the past (ref: no)		0.859	-
Schistosomiasis treatment, anytime in the past (ref: no)		0.396	-
Time since last Schistosomiasis treatment (ref: ≤ 20 years)		0.886	-
Intestinal parasites treatment, anytime in the past (ref: no)		0.589	-
Time since last intestinal parasites treatment (ref: ≤ 20 years)		0.555	-
To have contact with water from rivers, streams or lakes (ref: no)		0.988	1.002 (0.765-1.312)
To do the laundry with water from rivers, streams or lakes (ref: no)		0.464	-
To wash dishes with water from rivers, streams or lakes (ref: no)		0.701	-
To wash yourself with water from rivers, streams or lakes (ref: no)		0.890	-
To wash the children with water from rivers, streams or lakes (ref: no)		0.535	-

To swim in water from rivers, streams or lakes (ref: no)	0.126	-
To cross rivers, streams or lakes (ref: no)	0.212	-
To cook with water from rivers, streams or lakes (ref: no)	0.551	-
To fish with a net in rivers, streams or lakes (ref: no)	0.436*	-
To fish with a hook in rivers, streams or lakes (ref: no)	0.629	-
To use water from rivers, streams or lakes for agriculture activities (ref: no)	0.680	-
To use water from rivers, streams or lakes for religious activities (ref: no)	0.271*	-
To use water from rivers, streams or lakes for other activities (ref: no)	0.861	-
To use soap to do the laundry (ref: no)	0.348	-
To use soap to wash dishes (ref: no)	0.036	2.412 (1.034-5.624)
To use soap to wash hands (ref: no)	0.056	-
To use soap to wash yourself (ref: no)	0.715*	-
Blood in urine dipstick (ref: no)	0.034	1.452 (1.027-2.055)
Proteinuria in urine dipstick (ref: no)	0.364	-
Leukocytes in urine dipstick (ref: no)	0.618	-
Nitrites in urine dipstick (ref: no)	0.003	2.677 (1.351-5.306)
Schistosoma haematobium infection (ref: no)	0.005	1.823 (1.197-2.777)
Schistosoma mansoni infection (ref: no)	0.908	-
Ascaris lumbricoides (ref: no)	0.583	-
Hookworm (ref: no)	0.089*	-
Trichuris trichiura (ref: no)	0.999*	-

ref, reference class

*Bivariate analysis using Fisher`s exact test
